# SNP co-association and network analyses identify E2F3, KDM5A and BACH2 as key regulators of the bovine milk fatty acid profile

**DOI:** 10.1038/s41598-017-17434-7

**Published:** 2017-12-11

**Authors:** Sara Pegolo, Christos Dadousis, Núria Mach, Yuliaxis Ramayo-Caldas, Marcello Mele, Giuseppe Conte, Stefano Schiavon, Giovanni Bittante, Alessio Cecchinato

**Affiliations:** 1 Department of Agronomy, Food, Natural resources, Animals and Environment (DAFNAE), University of Padua, Viale dell’Università 16, 35020 Legnaro Padua, Italy; 2 Animal Genetics and Integrative Biology unit (GABI), INRA, AgroParisTech, Université Paris-Saclay, 78350 Jouy-en-Josas, France; 3 Animal Breeding and Genetics Program, Institute for Research and Technology in Food and Agriculture (IRTA), Torre Marimon, Caldes de Montbui, 08140 Spain; 4 Department of Agriculture, Food and Environment, University of Pisa, Via del Borghetto 80, 56124 Pisa, Italy

## Abstract

The fatty acid (FA) profile has a considerable impact on the nutritional and technological quality of milk and dairy products. The molecular mechanism underlying the regulation of fat metabolism in bovine mammary gland have been not completely elucidated. We conducted genome-wide association studies (GWAS) across 65 milk FAs and fat percentage in 1,152 Brown Swiss cows. In total, we identified 175 significant single nucleotide polymorphism (SNPs) spanning all chromosomes. Pathway analyses revealed that 12:0 was associated with the greatest number of overrepresented categories/pathways (e.g. mitogen-activated protein kinase (MAPK) activity and protein phosphorylation), suggesting that it might play an important biological role in controlling milk fat composition. An Associated Weight Matrix approach based on SNP co-associations predicted a network of 791 genes related to the milk FA profile, which were involved in several connected molecular pathways (e.g., MAPK, lipid metabolism and hormone signalling) and undetectable through standard GWAS. Analysis of transcription factors and their putative target genes within the network identified *BACH2*, *E2F3* and *KDM5A* as key regulators of milk FA metabolism. These findings contribute to increasing knowledge of FA metabolism and mammary gland functionality in dairy cows and may be useful in developing targeted breeding practices to improve milk quality.

## Introduction

Fatty acids (FAs) are energy substrates and important components of the cell membrane in the form of phospholipids. Their biological activities influence cellular and tissue metabolism and function, and signals responsiveness, consequently affecting health and disease risk^1^.

The nutritional and technological quality of milk is largely influenced by the quantity of milk fat and the fatty acid profile^2^. For instance, milk and dairy products are among the major sources of saturated fatty acids (SFAs) in the human diet. Epidemiological and clinical studies suggested that dietary SFAs are associated with a higher risk of cardiovascular disease, leading to a public health recommendation to decrease SFA intake^3^. By altering the milk fat composition, it may be possible to reduce SFA consumption without dietary intervention and changes in eating habits^4^. However, the quality of the evidence on which dietary recommendations are based has been recently challenged and it has been suggested that it would be preferable to consider the biological action of individual FAs rather than large FA groups^5^. Bovine milk contains, in fact, several bioactive FA such as butyric acid (4:0), palmitoleate (16:1 *c9*); oleic acid (18:1 *c9*), vaccenic acid (18:1 *t11*) and conjugated linoleic acid (CLA, 18:2 *c9*, *t11*)^6^.

Various factors can influence the milk FA composition, including stage of lactation, physiological state and feeding as well as the animal’s genetic background^7^. Several studies have shown the existence of heritable variations in the FA profile of bovine milk^8,9^, while genetic correlations among milk FA traits have been also estimated and biologically interpreted^9,10^. Furthermore, single nucleotide polymorphisms (SNPs) located in genes underlying quantitative trait loci for fat yield or content, such as *SREBP1*, *SCD1*, *DGAT1* and *ABCG2* ^11–13^, and in other genes involved in lipid biosynthesis or metabolism have been shown to affect the milk FA profile in different bovine breeds^14–16^. Genome-wide association studies (GWAS) have also shown several genomic regions to be significantly associated to the milk FA profile^17–19^, supporting the finding that milk fat synthesis and secretion are coordinated by a complex network of interrelated genes. The availability of data on the genetic parameters of individual FAs and identification of polymorphisms affecting their contents provide useful information for developing breeding selection strategies aimed at obtaining a milk FA profile more beneficial to human health.

Studies are available which investigated gene expression changes occurring in the bovine mammary gland across lactation^20,21^, as well as focused on the expression of lipid-related genes^22,23^ and on the discovery of genetic variants functionally implicated in the regulation of milk fat^24^. Therefore, several loci associated with milk FA composition have been identified and the knowledge about the role of genes driving milk fat synthesis in the bovine mammary gland has been significantly advanced. Despite that, the functional consequences of variants, including the complex molecular interplay of signal transduction, transcriptional, post-transcriptional and metabolic events underlying the regulation of milk fat synthesis and secretion in the bovine mammary gland, are still far to be extensively elucidated. A greater understanding of how genes or gene variants associated with milk FA composition exert their molecular effects could improve our knowledge of the physiological and cellular adaptations required for the synthesis and secretion of milk fat in ruminants, advance our understanding of tissue function beyond the well-known biochemical pathways and can improve the quality of milk and dairy products destined for human consumption.

The integration of GWAS analysis with gene-set enrichment and network analyses turned out to be a valid means to extrapolate additional biological information and investigate the functional relationships among sets of genes, that individually explain only a relatively small part of phenotypic variation and therefore cannot be identified by GWAS due to the stringent significance threshold^25^. These approaches have been recently used to explore bovine reproductive^26,27^ and productive traits^28,29^, and milk fat composition (including 10 FA traits)^19^ and have provided new insights into the biological pathways, complex gene interactions and key regulators affecting the investigated traits. Recently, an approach named Association Weight Matrix and based on SNP co-associations has been also proposed as a tool to exploit the results of GWAS and, combined with network inference algorithms, build gene networks to infer regulatory and functional interactions among genes^30^. Therefore, our hypothesis is that the milk FA associated alleles exert their effects by influencing transcription of the closest genes through multiple mechanisms. The aims of this study, therefore, were i) to perform a GWAS analysis to identify genomic regions associated to 65 FA traits and the fat percentage in bovine milk; ii) to characterize the regulatory mechanisms of the loci identified to understand the molecular and biological functions involved in the regulation of milk fat content and composition through pathway analysis; iii) to use an AWM approach to build gene networks for milk FA composition and to identify key transcription factors (TFs) regulating the synthesis and secretion of milk fat in dairy cows.

## Results

### GWAS analysis

Summary statistics and genomic heritabilities for the 65 FA traits and the fat percentage in bovine milk are reported in Tables 1 and 2. Heritability estimates varied from low (<0.10; e.g., 14:0 and 18:1*c9*) to moderate (up to 0.35; e.g., 16:0) with some individual FAs having values close to 0 (13:0, 22:0, 16:1*t9*, 17:1*c9*, 18:1*t4*, 18:2*t11*,*c15*, 18:3*c9*,*t11*,*c15*).

**Table 1:** Descriptive statistics, additive genetic variance $({\sigma }_{a}^{2})$ and genomic heritability $({h}^{2})$ for fat percentage and individual milk fatty acids (n = 1,152, Brown Swiss cows).

	Mean^1^	SD^1^	σ_a_^2^	h^2^	#SNP^2^
Fat, %	4.23	0.73	0.056	0.102	5
Individual FA, g/100 g fatty acids					
*SFA*					
4:0	3.46	0.91	0.031	0.109	—
6:0	2.15	0.39	0.005	0.087	4
8:0	1.35	0.23	0.005	0.152	1
10:0	3.17	0.63	0.044	0.197	6
11:0	0.06	0.04	<0.001	0.050	5
12:0	3.72	0.75	0.073	0.235	5
13:0	0.11	0.04	<0.001	<0.001	2
13:0 *iso*	0.06	0.04	0.089	0.075	—
14:0	12.08	1.56	0.089	0.075	4
14:0 *iso*	0.17	0.05	<0.001	0.134	8
15:0	1.19	0.24	0.003	0.094	7
15:0 *iso*	0.28	0.08	<0.001	0.099	2
15:0 *ante*	0.53	0.12	0.001	0.223	3
16:0	30.54	3.72	2.297	0.345	3
16:0 *iso*	0.32	0.09	0.001	0.149	4
17:0	0.54	0.12	<0.001	0.090	4
17:0 *iso*	0.32	0.08	<0.001	0.098	7
17:0 *ante*	0.42	0.09	0.001	0.145	2
18:0	8.95	1.87	0.471	0.226	2
20:0	0.13	0.04	<0.001	0.109	1
22:0	0.06	0.03	<0.001	<0.001	—
24:0	0.04	0.02	<0.001	0.025	6
*MUFA*					
10:1*c9*	0.33	0.09	0.001	0.260	5
14:1*c9*	1.08	0.32	0.015	0.286	14
16:1*c9*	1.21	0.32	0.012	0.184	—
16:1*t9*	0.06	0.03	<0.001	<0.001	1
17:1*c9*	0.20	0.08	<0.001	<0.001	7
18:1*t4*	0.03	0.02	<0.001	<0.001	2
18:1*t6-t8*	0.21	0.07	<0.001	0.081	2
18:1*t9*	0.18	0.06	<0.001	0.083	—
18:1*t10*	0.29	0.10	<0.001	0.073	1
18:1*t11 (VA)*	1.20	0.38	0.006	0.147	3
18:1*c9*	18.33	3.21	0.438	0.071	3
18:1*c12*	0.24	0.10	0.001	0.167	4
18:1*t16*	0.24	0.08	<0.001	0.089	4
20:1*c9*	0.11	0.04	<0.001	0.055	6
*PUFA*					
18:2*c9*,*t11 (RA)*	0.65	0.22	0.003	0.184	3
18:2*t11*,*c15*	0.10	0.08	<0.001	<0.001	2
18:2*c9*,*c12*	2.04	0.60	0.017	0.163	1
18:3*c9*,*c12*,*c15*	0.56	0.17	0.002	0.218	—
18:3*c9*,*t11*,*c15*	0.04	0.03	<0.001	<0.001	1
20:3*c8*,*c11*,*c14*	0.10	0.06	<0.001	0.046	2
20:4*c5*,*c8*,*c11*,*c14*	0.13	0.05	<0.001	0.220	2
20:5*c5*,*c8*,*c11*,*c14*,*c17*	0.05	0.02	<0.001	0.009	—
22:4*c7*,*c10*,*c13*,*c16*	0.03	0.02	<0.001	0.065	4
22:5*c7*,*c10*,*c13*,*c16*,*c19*	0.08	0.03	<0.001	0.049	3

^1^From Pegolo *et al*. (2016).

SD: standard deviation; σ_a_^2^ = genetic variance; h^2^: genomic heritability;

^2^#SNP: number of significant SNP (5 × ${10}^{-5}$) for each trait

SFA: saturated fatty acids; MUFA: mono-unsaturated fatty acids; PUFA: polyunsaturated fatty acids; VA: vaccenic acid; RA: Rumenic acid.

**Table 2:** Descriptive statistics, additive genetic variance and genomic heritability for groups of fatty acids and unsaturation indices (n = 1,152, Brown Swiss cows).

	Mean^1^	SD^1^	σ_a_^2^	h^2^	#SNP^2^
Group of fatty acids, g/100 g fatty acids					
SFA	69.63	4.11	1.366	0.156	3
MUFA	24.24	3.46	0.737	0.105	3
PUFA	3.78	0.79	0.055	0.253	2
SCFA	10.52	1.72	0.156	0.121	7
MCFA	52.81	5.26	2.093	0.194	2
LCFA	34.38	5.14	1.498	0.122	3
BCFA	2.08	0.41	0.013	0.262	4
n-3 fatty acids	0.69	0.20	0.003	0.199	4
n-6 fatty acids	2.31	0.65	0.020	0.167	2
n6/n3 ratio	3.53	1.18	0.034	0.108	5
* Trans* fatty acids	2.22	0.53	0.019	0.195	4
* Trans* fatty acids 18:1	2.16	0.52	0.018	0.193	3
Unsaturation index, %					
10:1/(10:0+10:1)	9.54	2.00	<0.001	0.224	8
14:1/(14:0+14:1)	8.16	2.04	<0.001	0.336	19
16:1/(16:0+16:1)	3.83	0.90	<0.001	0.176	—
18:1/(18:0+18:1)	67.22	4.32	<0.001	0.257	5
RA/(RA+VA)	34.98	5.53	<0.001	0.109	1

^1^From Pegolo *et al*. (2016).

SD: standard deviation; σ_a_^2^ = genetic variance; h^2^: genomic heritability;

^2^#SNP: number of significant SNP (5 × ${10}^{-5}$) for each trait

SFA: saturated fatty acids; MUFA: mono-unsaturated fatty acids; PUFA: polyunsaturated fatty acids; SCFA: short-chain fatty acids; MCFA: medium-chain fatty acids; LCFA: long-chain fatty acids; BCFA: branched-chain fatty acids; OCFA: odd-chain fatty acids; RA: rumenic acid; VA: vaccenic acid

SCFA included the 4:0, 6:0, 8:0 and C10:0 fatty acids; MCFA included all linear fatty acids from 11:0 to 16:1; LCFA included all linear fatty acids from 17:0 to 24:0; *trans* fatty acids included all trans fatty acids; *trans* fatty acids 18:1 included all trans isomers of 18:1.

**Figure 1:**
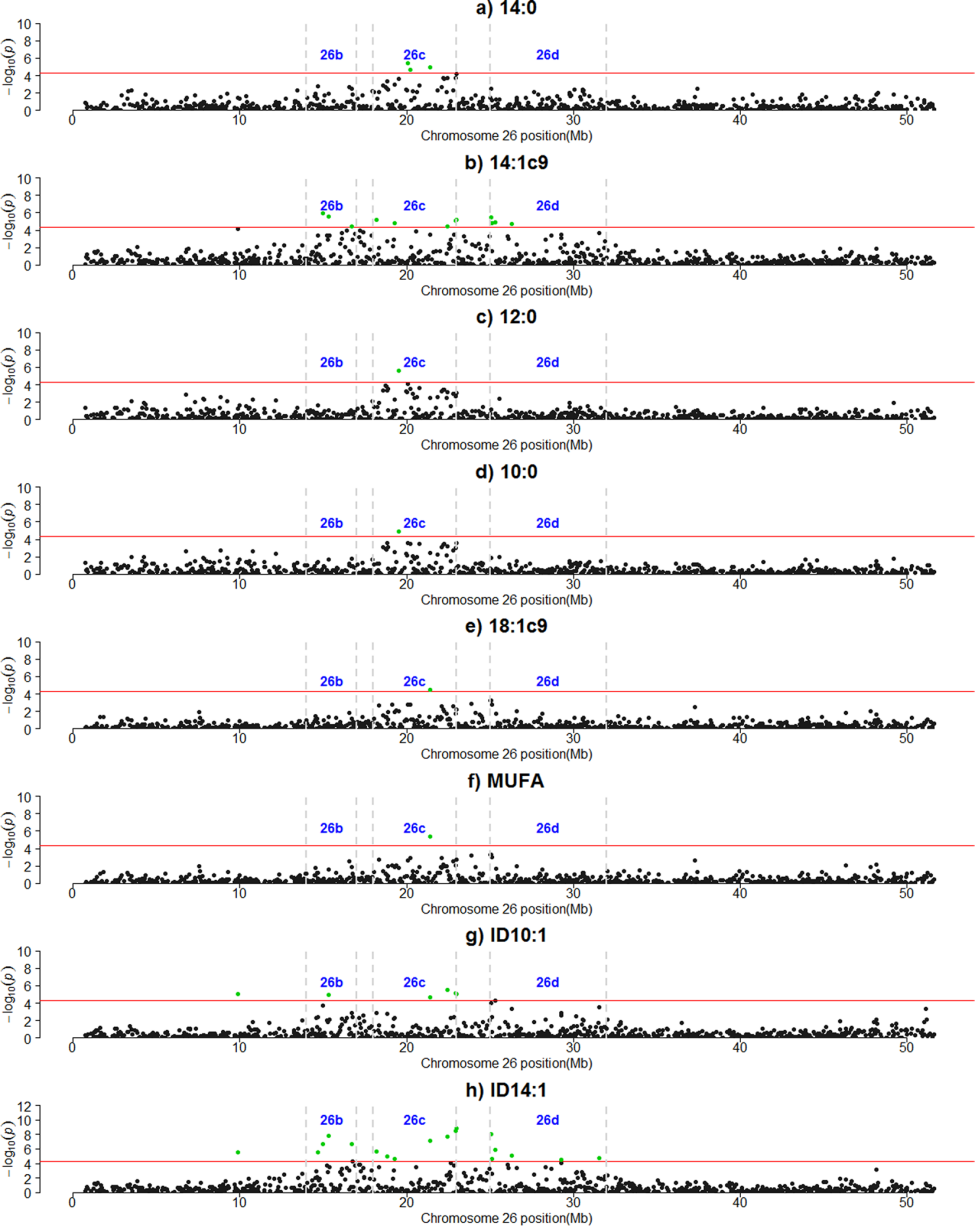
Manhattan plots of the genome-wide association studies on *Bos taurus* autosome 26 (BTA26). (**a**) 14:0; (**b**) 14:1*c9*; (**c**) 12:0; (**d**) 10:0; (**e**)18:1*c9*; (**f**) MUFA; (**g**) ID10:1; (**h**) ID14:1. The red horizontal lines indicate a −log10 (*P-*values) of 4.30 (corresponding to *P*-value = 5 × 10^−5^). Regions 26b, 26c and 26d are highlighted for each trait.

The results of the GWAS analysis are summarized in Table 3 and Supplementary Table S1. A total of 175 significant SNPs (*P* < 5E-05) were identified across all *Bos taurus* autosomes (BTAs). Three SNPs had unknown positions on the genome. Fifty-seven FA traits and the milk fat percentage exhibited significant signals, some of which were shared among the traits. The *P*-values ranged from 4.94E-05 to 1.36E-09. Around 50% of significant SNPs were associated to more than 1 trait. The highest signals were detected on BTA26 (~22.98 Mbp) and on BTA8 (~3.66 Mbp). In particular, the marker ARS-BFGL-NGS-32553 located at 22,977,848 bp on BTA26 was significantly associated with the 14:1 index (*P* = 1.36E-09). Other strong signals associated with the 14:1 index were detected at 22,951,431 bp and at 22,446,047 bp, which corresponded to markers ARS-BFGL-NGS-39823 and BTB-00933928 (*P* = 1.95E-08 and *P* = 8.07E-08, respectively). These two markers are in high linkage disequilibrium with ARS-BFGL-NGS-32553 (r2 = 0.89 and 0.70, respectively). A total of 4 regions (i.e. windows of consecutive SNPs at ≤ 1 Mb distance interval), that were detected on BTA26, were associated with 8 traits. Region 26a corresponded to only 1 SNP (~9.87 Mbp), which was associated to both 10:1 and 14:1 indices (Table 3). Regions 26b, 26c and 26d contained multiple associated SNPs (Table 3 and Fig. 1). In particular, region 26b (~14.66–16.71 Mbp) included 4 SNPs associated to 14:1 index and 14:1*c9*. In the region 26c (~18.17–22.98 Mbp), signals were detected for 14:1 index, 10:1 index, 14:1*c9*, MUFAs, 18:1*c9*, 10:0, 12:0 and 14:0. Finally, 6 SNPs in region 26d (~25.09–31.58 Mbp) were associated to 14:1 index and 14:1*c9*. The highest signal on BTA8 (Hapmap40047-BTA-119117) was significantly associated to 18:1*t16*. The proportion of additive genetic variance explained by this SNP was 44.22% (Supplementary Table S1). We detected regions of consecutive SNPs (≥3) on BTA3, BTA5 and BTA13. In particular, a region containing 6 SNPs on BTA3 (~118.57–121.20 Mbp, region 3 g) was significantly associated with 17:0 *iso*. Two regions including 5 SNPs were detected on BTA5: region 5a (~10.37–10.79 Mbp) which showed significant associations with 11:0, 15:0 and odd-chain FAs (OCFAs); region 5e (~84.09–85.29 Mbp), which contained signals for 10:0, 12:0, SFAs, monounsaturated FAs (MUFAs), medium-chain FAs (MCFAs) and long-chain FAs (LCFAs). Five SNPs on BTA13 (region 13c, ~42.07–42.93 Mbp) were associated with short-chain FAs (SCFAs), 8:0, 10:1*c9* and 15:0 *ante*.

**Table 3:** Summary results of the genome-wide association analysis for milk fatty acid traits.

BTA^1^	#SNP	Interval, Mbp	P-value (range)	Top SNP name/rs number	Top SNP location, bp	Top SNP MAF	Trait^2^
1	1	—	3.75E–05	DPI-28/rs109146949	37046480	0.21	13:0
1	1	—	2.83E–05	Hapmap49149-BTA-39529/rs43253949	81271586	0.21	18:1c12
1	1	—	4.00E–05	ARS-BFGL-NGS-23253/rs43248299	89900842	0.06	14:1 index
1	1	—	1.37E–05	BTB-01748272/rs42864406	92171349	0.01	17:1*c9*
1	1	—	4.04E–05	BTA-49368-no-rs/rs41578200	121093829	0.48	n6/n3 ratio
1	1	—	1.20E–07	ARS-BFGL-NGS-42512/rs43003942	138452988	0.13	BCFA, 14:0iso, **16:0** ***iso***
1	1	—	1.34E–05	BTB-01839901/rs42951145	140996730	0.09	n6/n3 ratio
1	1	—	4.23E–06	ARS-BFGL-NGS-97531/rs110831311	148969868	0.07	18:1*t11*
2	2	129.054–130.511	(6.94E–06, 3.00E–06)	ARS-BFGL-NGS-56131/rs110614098	130511453	0.20	**OCFA**, 18:2*c9*,*t11*
3a	1	—	3.06E–05	ARS-BFGL-NGS-100336/rs109285212	13148505	0.01	n6/n3 ratio
3b	1	—	1.09E–05	ARS-BFGL-NGS-108225/rs109664220	45572779	0.01	17:1*c9*
3c	1	—	5.73E–06	Hapmap59096-rs29024776/rs29024776	49335664	0.01	17:1*c9*
3d	2	56.959–57.516	(8.50E–06, 3.49E–06)	ARS-BFGL-NGS-69251/rs208524162	57515766	0.01	17:1*c9*
3e	1	—	1.56E–05	BTB-00135284/rs43342803	72743814	0.01	OCFA
3 f	1	—	1.73E–05	ARS-BFGL-NGS-34260/rs43578470	78170557	0.06	10:1*c9*
3 g	6	118.567–121.204	(8.99E–06, 1.28E–06)	BTB-01730472/rs42844513	120283544	0.34	17:0*iso*
4	1	—	4.90E–06	Hapmap49725-BTA-72716/rs41653969	25155275	0.13	24:0
4	1	—	1.49E–05	ARS-BFGL-NGS-43812/rs110040170	84080999	0.03	n3
5a	5	10.336–10.789	(2.85E–05, 1.02E–05)	BTA-23621-no-rs/rs41607929	10735432	0.09	11:0, 15:0, **OCFA**
5b	1	—	7.18E–06	Hapmap30002-BTA-142983/rs110558219	26876852	0.01	18:1 index
5c	1	—	3.66E–05	ARS-BFGL-NGS-22065/rs110164442	41695035	0.19	18:0
5d	1	—	4.24E–05	BTA-73516-no-rs/rs41657461	48752237	0.33	18:0
5e	5	84.087–85.289	(2.00E–05, 4.58E–06)	ARS-BFGL-NGS-72008/rs109763804	85160180	0.35	10:0, **12:0**, SFA, MUFA, MCFA, LCFA
5 f	1	—	1.64E–05	ARS-BFGL-NGS-99256/rs109920572	104714350	0.37	**PUFA**, n6
5 g	1	—	3.15E–05	ARS-BFGL-NGS-91167/rs41565304	108721301	0.11	20:1*c9*
6a	1	—	1.11E–07	Hapmap46514-BTA-122322/rs42706774	1091047	0.01	20:4*c5*,*c8*,*c11*,*c14*, **CLA index**
6b	2	21.830–23.148	(1.28E–05, 6.17E–06)	ARS-BFGL-NGS-118959/rs42960052	21829670	0.18	18:1*t16*
6c	1	—	1.06E–05	Hapmap23862-BTC-069949/rs42974158	40530400	0.01	17:0*ante*
6d	1	—	2.80E–05	Hapmap38352-BTA-76628/rs41567758	70865694	0.04	18:2*t11*,*c15*
6e	1	—	2.31E–05	BTA-76070-no-rs/rs41651324	78598487	0.01	18:1*t6–8*
7	1	—	3.51E–07	ARS-BFGL-NGS-106506/rs110440837	8491850	0.17	BCFA, **15:0ante**
7	1	—	6.38E–06	ARS-BFGL-NGS-27096/rs42584535	34158112	0.03	18:1*c12*
7	1	—	7.80E–06	BTB-01848865/rs42957016	87008149	0.13	fat
7	1	—	3.05E–05	BTB-01862398/rs42974286	89421646	0.01	16:0
8a	1	—	2.53E–09	Hapmap40047-BTA-119117/rs42871459	3663959	0.01	18:1*t16*
8b	1	—	4.83E–05	ARS-BFGL-NGS-103495/rs109183089	9393962	0.28	15:0*ante*
8c	1	—	3.51E–05	BTA-109900-no-rs/rs41611360	22479821	0.24	16:0
8d	1	—	3.24E–05	Hapmap31882-BTA-80969/rs41607560	36781224	0.13	SFA
8e	1	—	2.82E–05	ARS-BFGL-NGS-66921/rs43557108	63901386	0.08	24:0
8 f	1	—	2.39E–05	ARS-BFGL-NGS-79292/rs109397331	76353681	0.03	20:3*c8*,*c11*,*c14*
8 g	1	—	1.58E–05	ARS-BFGL-NGS-25285/rs110783907	86501009	0.01	13:0
8 h	1	—	2.43E–05	BTB-00372235/rs43575220	102000000	0.12	18:1*c12*
8i	2	109.112–110.602	(3.87E–05, 1.59E–05)	Hapmap57174-rs29021038/rs29021038	109000000	0.03	**LCFA**, MCFA, MUFA, SFA, 17:1*c9*
9a	3	10.454–10.859	(8.76E–06, 4.69E–06)	Hapmap38633-BTA-83140/rs41592651	10858927	0.42	14:0*iso*, **24:0**
9b	1	—	3.67E–05	ARS-BFGL-NGS-82987/rs109263914	13141454	0.01	20:1*c9*
9c	1	—	3.71E–06	BTB-00389124/rs43593890	35036949	0.01	18:1 index
9d	1	—	4.02E–05	BTB-00396747/rs43601252	61292190	0.11	22:4*c7*,*c10*,*c13*,*c16*
9e	1	—	1.23E–06	BTB-00403297/rs43607069	91346194	0.42	fat
9 f	1	—	3.00E–05	ARS-BFGL-NGS-25581/rs109138962	97050334	0.34	17:0
9 g	1	—	9.79E–06	ARS-BFGL-NGS-72947/rs110309954	102301291	0.47	16:0
9 h	1	—	2.98E–05	ARS-BFGL-NGS-34445/rs108973184	104529625	0.38	**20:0**, TFA, TFA 18:1
10	1	—	3.29E–05	BTB-00415258/rs43621939	28680745	0.25	SCFA
10	1	—	3.79E–05	BTB-00424023/rs43627019	51775341	0.31	10:1 index
10	1	—	5.78E–06	BTA-111053-no-rs/rs43712043	98541920	0.40	15:0
11	1	—	1.99E–05	Hapmap36552-SCAFFOLD185127_16827/rs29014822	2767247	0.01	11:0
11	1	—	4.74E–05	ARS-BFGL-NGS-37630/rs109094066	46590323	0.20	SCFA
11	1	—	1.57E–05	ARS-BFGL-NGS-22364/rs110219158	72900738	0.04	20:3*c8*,*c11*,*c14*
12	2	81.184–82.564	(2.29E–05, 4.09E–06)	ARS-BFGL-NGS-113366/rs109738803	82564341	0.03	**18:1** ***t6–8***, 20:1*c9*
13a	1	—	4.36E–05	ARS-BFGL-NGS-93056/rs41679436	8880814	0.27	18:1*c12*
13b	1	—	1.71E–05	Hapmap51705-BTA-24478/rs41608666	21822462	0.03	15:0
13c	5	42.071–42.928	(3.21E–05, 4.71E–06)	ARS-BFGL-NGS-74106/rs109854819	42148059	0.01	**SCFA**, 8:0,10:1*c9*,15:0*ante*
13d	1	—	1.43E–05	ARS-BFGL-NGS-26401/rs109111862	56128139	0.02	22:5*c7*,*c10*,*c13*,*c16*,*c19*
13e	1	—	1.72E–05	BTB-00534445/rs41702380	60198826	0.01	22:5*c7*,*c10*,*c13*,*c16*,*c19*
13 f	1	—	6.76E–06	ARS-BFGL-NGS-36046/rs109889561	79523868	0.43	17:0
13 g	1	—	2.82E–05	ARS-BFGL-NGS-19988/rs110209373	83018263	0.06	17:0*ante*
14a	1	—	3.60E–05	Hapmap25183-BTC-049425/rs110642420	6910008	0.34	18:1*t4*
14b	1	—	2.82E–05	ARS-BFGL-NGS-114730/rs109081077	17378950	0.02	11:0
14c	1	—	8.25E–06	Hapmap25446-BTC-054694/rs110267285	26003598	0.46	fat
14d	1	—	2.38E–06	Hapmap50929-BTA-28833/rs42488778	40785938	0.02	14:0*iso*, **15:0** ***iso***
14e	2	57.146–58.849	(3.82E–05, 3.71E–05)	ARS-BFGL-NGS-18262/rs110902895	58848872	0.05	14:0*iso*, **15:0** ***iso***
15a	1	—	4.94E–05	ARS-BFGL-NGS-89820/rs41749553	9041018	0.38	22:4*c7*,*c10*,*c13*,*c16*
15b	2	22.960–23.155	(4.93E–05, 4.02E–05)	Hapmap49882-BTA-121007/rs41633877	22960231	0.48	17:0, 18:1*c9*, **LCFA**
15c	1	—	1.38E–06	ARS-BFGL-BAC-27778/rs110822031	41043816	0.01	**10:1** ***c9***, 14:1*c9*, 14:1 index
15d	1	—	1.33E–05	Hapmap43354-BTA-77081/rs41655008	70284345	0.41	**18:1** ***t10***, 15:0
16	1	—	1.74E–05	ARS-BFGL-NGS-87853/rs110144946	30262349	0.19	fat
16	1	—	2.74E–05	Hapmap41467-BTA-18750/rs42936429	34021608	0.23	n3
16	1	—	1.20E–05	BTA-38719-no-rs/rs41800166	37229378	0.11	11:0
16	1	—	3.13E–05	ARS-BFGL-NGS-77903/rs109547989	48694547	0.13	n3
16	1	—	2.38E–05	BTA-105815-no-rs/rs42703002	79192647	0.32	14:0*iso*
17	1	—	8.10E–06	Hapmap47504-BTA-111690/rs41567580	11231535	0.02	**18:3** ***c9***,***c12***,***c15***, n3
17	1	—	2.79E–05	Hapmap42781-BTA-105847/rs41611446	20593969	0.32	18:1*t11*
17	1	—	2.60E–05	Hapmap41708-BTA-99722/rs41596865	61624831	0.01	18:1*t4*
17	1	—	3.78E–05	ARS-BFGL-NGS-13495/rs42392402	69267462	0.07	24:0
18	1	—	4.84E–05	ARS-BFGL-NGS-116944/rs110779574	53071113	0.13	20:1*c9*
19a	1	—	5.83E–06	ARS-BFGL-NGS-107289/rs110773010	10305065	0.13	*18:1t11*, **TFA**,TFA 18
19b	2	38.366–39.202	(3.25E–05, 2.08E–05)	ARS-BFGL-NGS-20183/rs109740434	38365974	0.39	22:4*c7*,*c10*,*c13*,*c16*
19c	1	—	1.36E–05	UA-IFASA-6210/rs41579737	59715027	0.11	OCFA
19d	3	61.585–62.830	8.28E–06	ARS-BFGL-NGS-31729/rs110634188	62829939	0.42	10:1*c9*,18:1*c9*, **20:1** ***c9***
20a	2	36.757–37.839	(7.31E–06, 1.13E–06)	ARS-BFGL-NGS-5430/rs110515218	37838938	0.08	18:1 index
20b	1	—	4.66E–05	Hapmap26422-BTA-148751/rs110477372	40754324	0.01	17:0
20c	2	61.402–61.765	(4.44E–05, 2.80E–05)	ARS-BFGL-NGS-99194/rs110456601	61764939	0.08	**24:0**, 18:2*c9*,*c12*
20d	1	—	9.33E–06	ARS-BFGL-NGS-103163/rs110692744	68933034	0.38	10:1*c9*
20e	1	—	2.56E–05	ARS-BFGL-NGS-60835/rs110404528	71271028	0.42	14:1*c9*
21a	1	—	4.06E–05	Hapmap39215-BTA-105710/rs41617177	4706119	0.27	22:5*c7*,*c10*,*c13*,*c16*,*c19*
21b	1	—	2.76E–05	ARS-BFGL-NGS-119424/rs109012245	12684633	0.02	n6
21c	1	—	1.75E–05	Hapmap59970-rs29026939/rs29026939	42413671	0.28	**6:0**, SCFA
21d	2	63.985–64.085	(4.12E–05, 1.67E–06)	ARS-BFGL-NGS-43652/rs109547826	64085350	0.15	BCFA
22a	1	—	3.13E–05	ARS-BFGL-NGS-19546/rs109794490	21547900	0.01	18:3*c9*,*t11*,*c15*
22b	1	—	3.10E–06	ARS-BFGL-NGS-20317/rs109201435	29854903	0.02	17:1*c9*
22c	3	46.782–47.365	(2.42E–05, 6.60E–06)	ARS-BFGL-NGS-8294/rs42011605	46781645	0.10	6:0
22d	1	—	2.00E–06	ARS-BFGL-NGS-82789/rs42286369	58458470	0.10	n6/n3 ratio
23	1	—	2.00E–05	Hapmap40178-BTA-55802/rs41641235	21748514	0.36	18:1*t16*
23	1	—	8.29E–06	ARS-BFGL-NGS-109297/rs110759282	36139914	0.07	**15:0**, OCFA
24	1	—	4.20E–05	ARS-BFGL-NGS-19695/rs110816279	14841074	0.02	14:0*iso*
24	1	—	2.11E–05	ARS-BFGL-NGS-104621/rs109735501	48334377	0.13	20:1*c9*
25	1	—	1.68E–05	Hapmap29767-BTC-015734/rs109968431	2084697	0.10	16:0*iso*
25	1	—	4.53E–06	ARS-BFGL-NGS-108964/rs110840614	4566007	0.02	18:1 index
25	1	—	2.47E–05	Hapmap43893-BTA-60736/rs41645892	9862811	0.19	17:0*iso*
25	1	—	3.14E–07	BTA-118965-no-rs/rs42074723	32648759	0.14	**18:2** ***c9***,***t11***, PUFA
26a	1	—	3.05E–06	ARS-BFGL-NGS-13746/rs110924756	9866940	0.13	10:1 index, **14:1 index**
26b	4	14.655–16.708	(2.71E–06, 1.67E–08)	ARS-BFGL-NGS-43432/rs110578080	15336560	0.28	**14:1 index**, 14:1*c9*
26c	9	18.170–22.978	(2.52E–05, 3.46E–09)	ARS-BFGL-NGS-39823/rs42089958	22951431	0.09	**14:1 index**,10:1 index, 14:1*c9*, MUFA, 18:1*c9*, 10:0, 12:0, 14:0
26d	6	25.088–31.577	(1.61E–05, 9.05E–09)	ARS-BFGL-NGS-118712/rs42095154	25088146	0.19	**14:1 index**, 14:1*c9*
27	1	—	6.56E–07	ARS-BFGL-NGS-87845/rs109663833	42118037	0.03	fat
28	1	—	2.52E–05	ARS-BFGL-NGS-118662/rs110810782	2947166	0.01	18:2*c9*,*t11*
29	1	—	4.39E–05	ARS-BFGL-NGS-67720/rs42636308	17876279	0.06	22:4*c7*,*c10*,*c13*,*c16*
29	1	—	4.60E–06	BTA-65012-no-rs/rs42168039	19966479	0.10	14:0*iso*
U^3^	1	—	5.30E–06	ARS-BFGL-NGS-102692/rs43587199	0	0.46	16:0*iso*
U^3^	1	—	3.22E–05	BTB-00021257/ rs43232419	0	0.38	18:2*t11*,*c15*
U^3^	1	—	4.90E–05	Hapmap43001-BTA-63377/ rs41650170	0	0.19	16:1*t9*

^1^BTA = *Bos taurus* autosome; #SNP = number of the single nucleotide polymorphisms significantly associated to the trait; Interval: The region on the chromosome spanned among the significant SNP(s) (in Mb); P-value (range) = The P-value of the highest significant SNP adjusted for genomic control and the range of the P-values when multiple SNP were significantly associated to one trait; Top SNP location (bp) = position of the highest significant SNP on the chromosome in base pairs on UMD3.1 (http://www.ensembl.org/index.html); Top SNP MAF = minor allele frequency of the top SNP.

^2^OCFA: odd-chain fatty acids; BCFA: branched-chain fatty acids; SFA: saturated fatty acids; MUFA: monounsaturated fatty acids; PUFA: polyunsaturated fatty acids; SCFA: short-chain fatty acids; LCFA: long-chain fatty acids; TFA: trans fatty acids; TFA 18:1: trans fatty acids 18:1; CLA: conjugated linoleic acid. SCFA included the 4:0, 6:0, 8:0 and C10:0 fatty acids; MCFA included all linear fatty acids from 11:0 to 16:1; LCFA included all linear fatty acids from 17:0 to 24:0; trans fatty acids included all trans fatty acids; trans fatty acids 18:1 included all trans isomers of 18:1.

The trait with the highest P-value in each genomic region is bolded.

^3^U:Undefined chromosome and position on the genome.

### Pathway analyses

**Figure 2:**
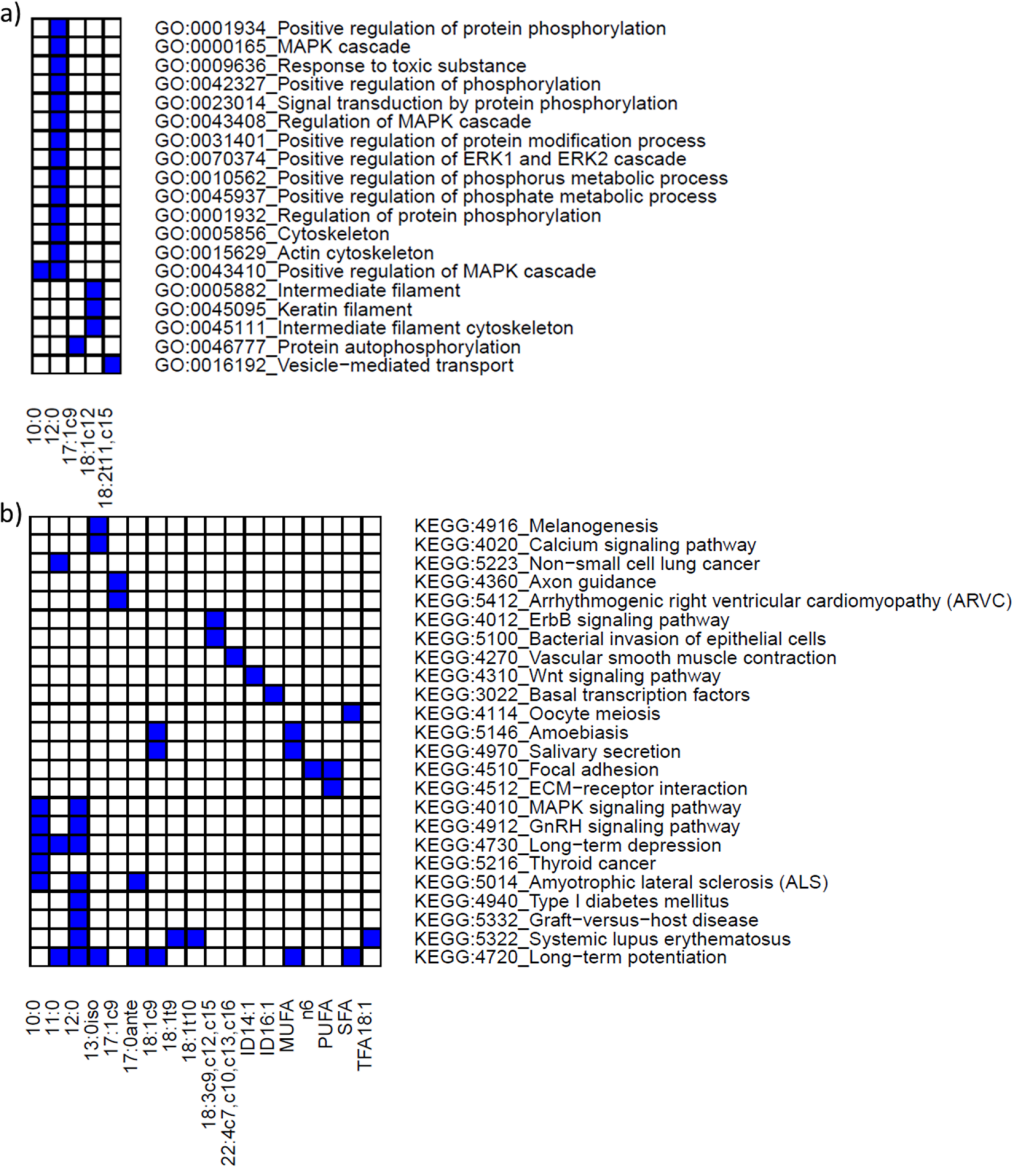
Distribution of the significantly enriched terms/pathways using genes associated to the fatty acid traits. The SNP (*P* < 0.05) were assigned to genes if they were located within the gene or in a flanking region of 15 kb up- and downstream of the gene using the biomaRt R package. For mapping, the Ensembl *Bos taurus* UMD3.1 assembly was used as reference. Gene-set enrichment analysis was carried out using the *goseq* R package. Only the traits showing significantly enriched terms are reported (FDR < 0.05). (**a**) GO terms; (**b**) KEGG-pathways. ID14:1: 14:1/(14:1+14:0); ID16:1: 16:1/(16:0+16:1); MUFA: monounsaturated fatty acids; PUFA: polyunsaturated fatty acids; SFA: saturated fatty acids; TFA: *trans* fatty acids.

In the pathway analyses, for each trait 850 significant SNPs *(P* < 0.05) were on average assigned to 700 genes, which were mined using the Bioconductor package goseq^31^ to reveal the biological processes connected with the milk FA synthesis and metabolism in the bovine mammary gland. Significantly enriched GO terms and KEGG pathways (*q* < 0.05) were found for the profile of 20 out of the 65 FA traits in milk (Fig. 2, Supplementary Table S2). In particular, the genes associated with 12:0 presented the highest number of overrepresented categories/pathways, suggesting a large degree of genetic control for this FA (Fig. 2a). For instance, we observed a high association between the 12:0 content in milk and the regulation of mitogen-activated protein kinase (MAPK) activity, e.g., MAPK cascade (*q* = 7.73E-06), MAPK signalling pathway (*q = *0.00046), positive regulation of extracellular signal-regulated kinase (ERK) 1 and ERK2 cascade (*q* = 0.00017), and positive regulation of protein phosphorylation (*q* = 8.70E-06; Fig. 2a). The intermediate filament together with the intermediate filament cytoskeleton appeared to be substantially enriched by 18:1*c12 (q = *3.99E-06 and *q = *8.34E-06, respectively), while vesicle-mediated transport was generally enhanced by 18:2*t11*,*c15*-related genes (*q* = 1.57E-05) (Fig. 2a).

Similar to GO terms, KEGG analysis indicated that the 12:0-related genes were involved in the GnRH signalling pathway, MAPK signalling, type I diabetes mellitus and inflammation processes such as graft-versus-host disease and systemic lupus erythematosus (Fig. 2b). The same analysis indicated that the systemic lupus erythematosus pathway was also enriched for 18:1*t9*, 18:1*t10*, TFA and TFA18:1. The oocyte meiosis pathway was ranked at the top of the KEGG list of the most impacted functions for SFA (*q* = 0.00017; Fig. 2b), whereas the ErbB signalling pathway and bacterial invasion of epithelial cells was clearly induced by 18:3*c9*,*c12*,*c15*-related genes. Basal transcription factors appeared to be greatly impacted by the 16:1 index-related genes (*q* = 0.00020; Fig. 2b).

### AWM matrix construction and gene networks

**Figure 3:**
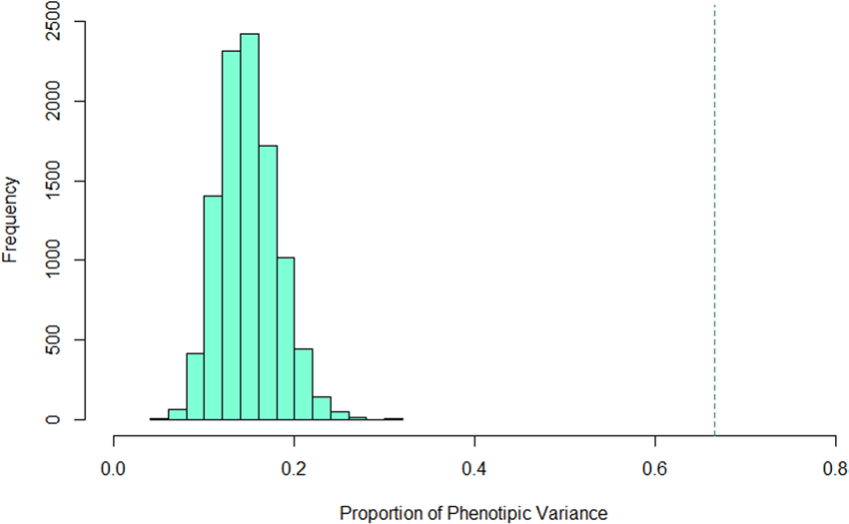
Proportion of phenotypic variance explained by 1575 randomly selected SNP (10,000 replicates). The dashed green vertical line represents the proportion of phenotypic variance explained by the SNPs include in the AWM.

**Figure 4:**
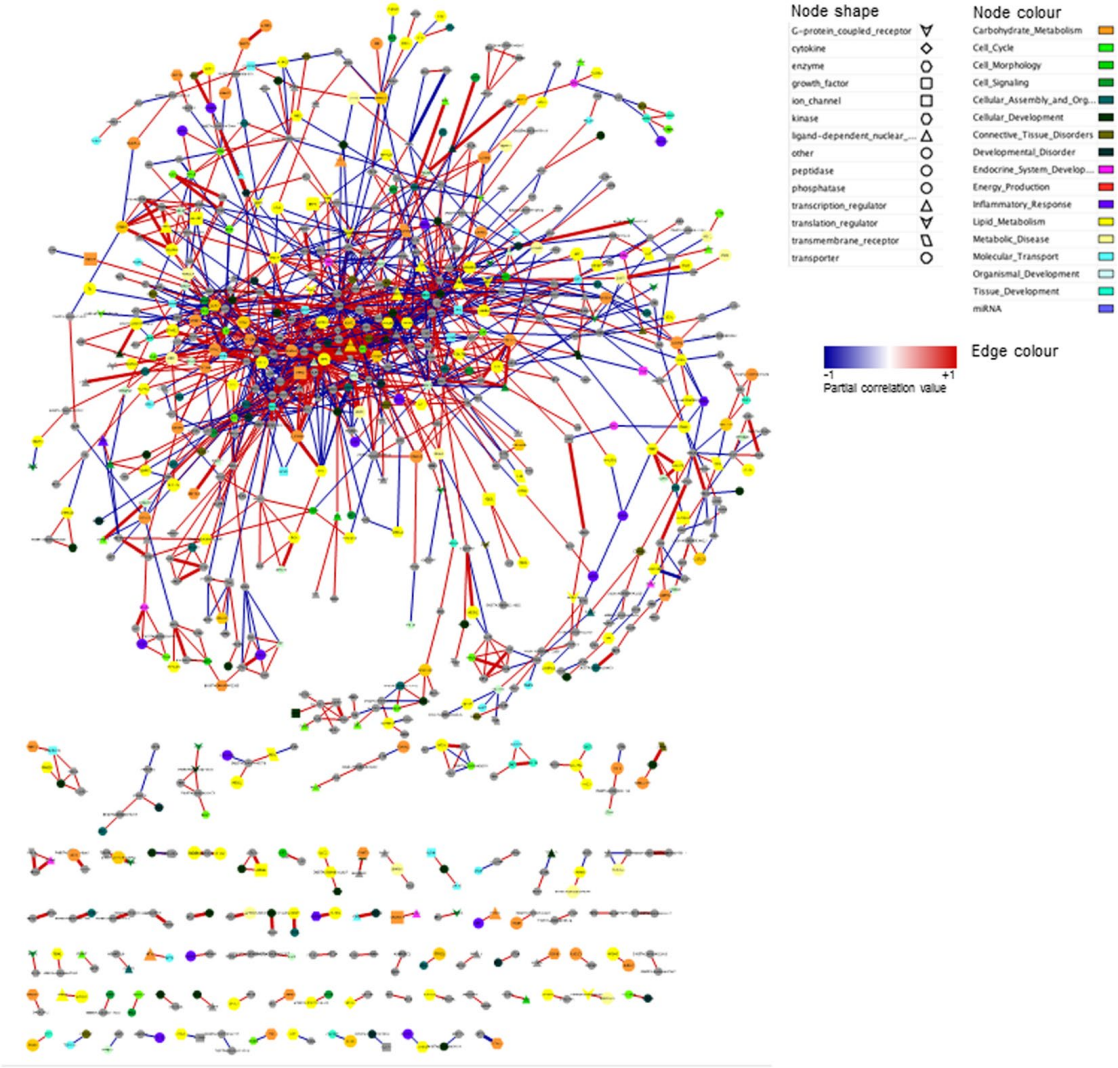
Regulatory network of the genes significantly associated with fatty acid profiles in bovine milk. In the network, every node represents a gene, whereas every edge connecting two nodes represents a significant interaction (correlation value ≥|0.80|). The nodes shape indicates whether the node is a transcription factor (triangles), a miRNA (hexagon), a metabolite (round rectangle), a membrane receptor (rectangle), a transporter (parallelogram), or other type of genes (ellipses). Information of molecule type was obtained using Ingenuity Pathway Analysis (IPA; version 5.5, Ingenuity Systems, USA). The list of identified TF was updated with that reported by^33^. The node colour represents the biological function of the gene, according to IPA. The edge colour intensity indicates the level of the association: red = positive correlation - and blue = negative correlation between two nodes. The width of the edge indicates the value of the correlation; a wider edge corresponds to a higher correlation in absolute value.

We retained 15,277 annotated SNPs out of the 37,568 SNPs for AWM matrix construction analyses. After applying a series of filtering steps (see Material and Methods), 1,575 SNPs, corresponding to 1,575 unique genes, were used to build the AWM matrix. Hierarchical clustering of traits evidenced 2 clusters which best described the FA profiles according to similarities in their origins or in their metabolic processes, e.g., alpha-linolenic acid (18:3*c9*,*c12*,*c15*) clustered with the n3 FAs, and 6:0 with the SCFAs (Supplementary Fig. S1). Correlations across AWM rows were used to predict gene associations and build a network in which each node represented a gene and each edge a significant interaction. In total, 27,050 significant edges between 1,575 nodes were identified based on the Partial Correlation coefficient with Information Theory (PCIT) algorithm. The SNPs detected with the AWM approach explained 67% of the phenotypic variance for 12:0, which was significantly larger (*P* < 0.001) than the variance explained by the same number of randomly selected SNPs (10,000 replicates) (Fig. 3). After applying the gene network reduction strategy (filtering for sparse correlations ≥ |0.80|), we obtained a network with 1,628 significant interactions and 791 nodes (Fig. 4). The node degree followed an approximate power law distribution (R^2^ = 0.933; *y* = −1605*x*379), which suggests that the reduced network was scale-free and that a few gene nodes acted as hubs with a large number of links to other gene nodes. Analysis of other network topological parameters, e.g., node closeness, eccentricity and betweenness (network centrality indices), showed that some of the genes involved in lipid metabolism, such as *INTS8*, *SACM1L*, *AGMO* and *PTGR1*, might be relevant to the SNP co-association network. Table 4 summarizes the 10 most highly-connected nodes based on significant interactions (node degree) and Supplementary Table S3 shows their positions in close proximity to the QTLs related to milk fat or milk FAs deposited in the Cattle QTL database^32^. As the SNP marker Hapmap54104-rs29010930 was located ~ 2 kb up-stream of *CNNM1* (at ~20.3 Mb on BTA26), we investigated whether this SNP affected the transcriptional regulation of *CNNM1* by comparing genomic regions up-stream of *CNNM1* across different species using mVISTA. The results revealed the presence of a cluster of conserved non-coding elements (CNEs) surrounding Hapmap54104-rs29010930 (Supplementary Fig. S2).

**Table 4:** Description of the ten most connected nodes in the co-association network*.

SNP/Gene	Illumina Chip SNP/rs number	Ap^1^	Connections	Functional consequence
*CNNM1*	Hapmap54104-rs29010930/ rs29010930	16	45	Upstream variant 2 kb
*MED21*	ARS-BFGL-NGS-110407/ rs109351348	12	45	Intron variant
*SSPN*	Hapmap49290-BTA-74411/ rs41652667	22	43	Inter-genic variant
FBXO7	ARS-BFGL-NGS-76692/ rs110166704	10	41	Downstream variant 500 bp, utr variant 3 prime
*LRMP*	ARS-BFGL-NGS-110708/ rs109690396	18	35	Intron variant
*LARGE1*	Hapmap39452-BTA-94180/ rs41572821	15	34	Intron variant
*KRAS*	ARS-BFGL-NGS-72008/ rs109763804	14	34	Inter-genic variant
*BCAT1*	ARS-BFGL-NGS-39913/ rs109168591	14	32	Intron variant
*BTRC*	BTB-00932332/rs42088972	12	32	Intron variant
*DNAH5*	ARS-BFGL-NGS-119072/ rs110338011	19	31	Intron variant

^1^Ap: associated phenotypes.

*Network represented in Fig. 4 which was obtained after filtering the complete PCIT-gene network for sparse correlation ≥|0.80|.

We used IPA to investigate the co-enriched functions/pathways and other biological features within the network. Enriched pathways included MAPK related pathways (e.g. “ERK/MPK signaling”, *P* = 8.32E-03; “GnRH signaling”, *P* = 1.55E-06), “Phosphatidylglycerol Biosynthesis II” (*P* = 6.76E-03); pathways related to lipid metabolism such as “Fatty acid activation”(*P* = 9.12E-03), “Adipogenesis pathway”(*P* = 1.91E-02), “Triacylglycerol Biosynthesis” (*P* = 8.51E-03), “Triacylglycerol degradation” (*P* = 3.02E-02), “PPAR signalling” (*P* = 1.38E-03) and “CDP-diacylglycerol Biosynthesis I” (*P* = 4.47E-03); pathways related to hormone signalling, e.g., “Pregnenolone Biosynthesis” (*P* = 1.91E-02). The top IPA computed networks showed that the genes in the networks were associated with “Cell Morphology, Cellular Function and Maintenance, Carbohydrate Metabolism” and “Immunological Disease, Inflammatory Disease, Inflammatory Response”. The full list of enriched pathways and computed networks is reported in Supplementary Table S4.

**Figure 5:**
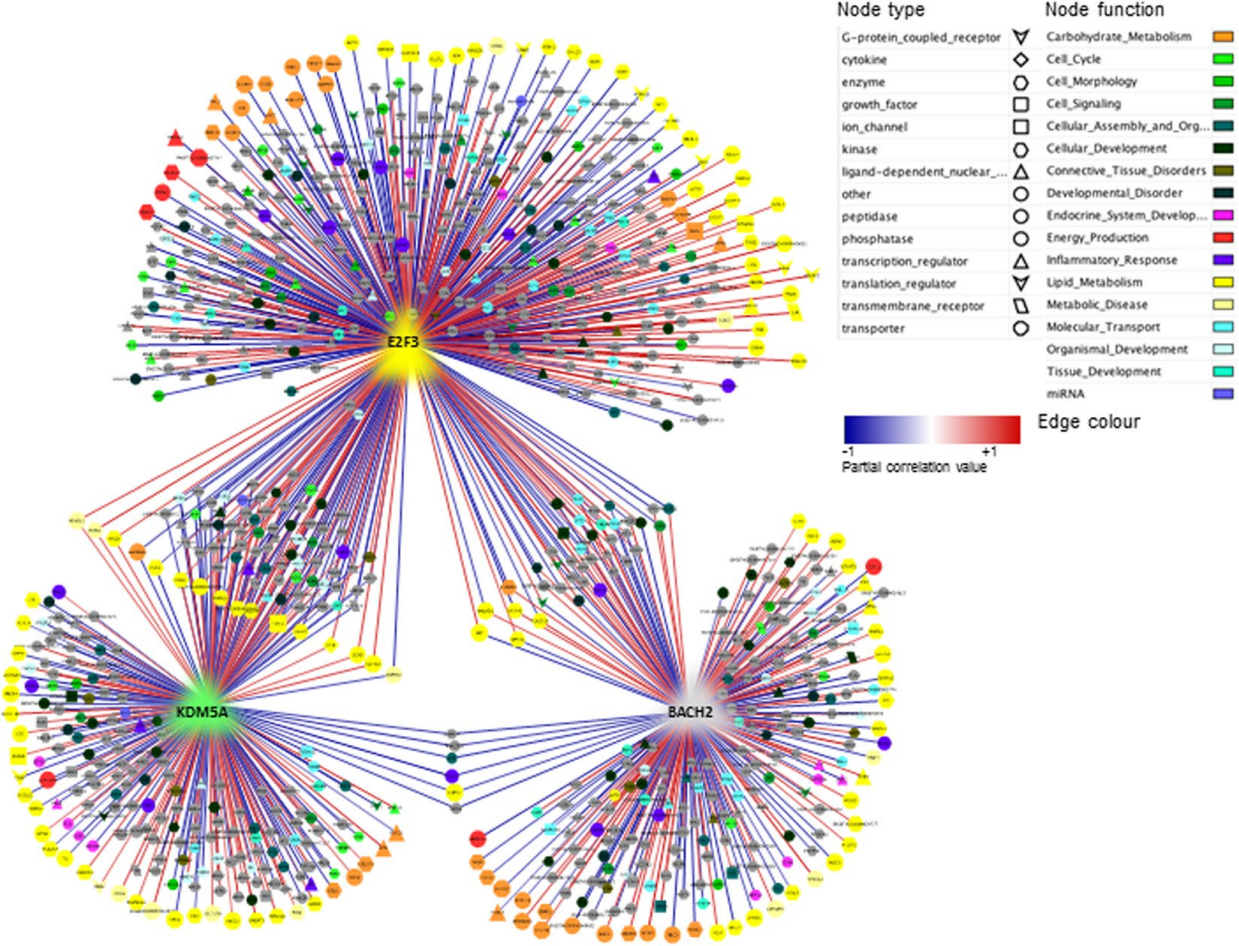
Activators and repressors of the regulatory network of genes associated with the bovine milk fatty acid profile. In the network, the best trio of transcription factors is showed: *E2F3*, *KDM5A* and *BACH2*. The nodes shape indicates whether the node is a transcription factor (triangles), a miRNA (hexagon), a metabolite (round rectangle), amembrane receptor (rectangle), a transporter (parallelogram), or other type of genes (ellipses). The node colour represents the biological function of the gene according to Ingenuity Pathway Analysis (IPA) annotation. The list of identified TF was updated with that reported by^33^. The edge colour intensity indicates the level of the association: red = positive correlation - and blue = negative correlation between two nodes.

In parallel, we have also generated a supplementary TF network by investigating the potential impact of the combination of one or more TFs on the expression of the other genes in the network with the lowest redundancy. Hence, we explored 383,306 possible combinations of TF trios among the 1,977 TFs identified by^33^. The analysis allowed us to identify BTB domain and CNC homolog 2 (*BACH2)*, E2F transcription factor 3 (*E2F)3*, and lysine demethylase 5 A *(KDM5A)* TFs, which controlled the transcription of 877 unique genes in our network (~56% of the genes in the AWM matrix) (Fig. 5) and which might play a pivotal role in orchestrating adaptations of the FA metabolism in the mammary gland. Functional analyses of these 877 target genes by ClueGo revealed the metabolic pathways to be the most highly impacted, of which lipid and carbohydrate metabolism were the most important. Among lipid metabolism, glycerophospholipid metabolism and sphingolipid metabolism were the most highly induced, followed by the MAPK activity-related pathways (e.g., the MAPK signalling pathway) and the GnRH signalling pathway. We also found overrepresentation of pathways related to reproduction (e.g., progesterone-mediated oocyte maturation) as well as the glutamatergic synapse and oxytocin signalling pathway. The full list of significantly enriched pathways and GO terms is presented in Supplementary Table S5.

In addition, to analyze in depth the TFs potentially controlling the network, we examined the putative binding sites within the promoter region of these TFs using the LASAGNA tool. Interestingly, the promoter region of *E2F3* and *KDM5A* was predicted to contain binding sites for several regulators of cholesterol and lipid metabolism such as *ARNT:AhR*, *HNF4A*, *CREB1*, *SP1*, *MAFB* and *PPARG*:*RXRA* (Supplementary Table S6).

## Discussion

### GWAS results

We have reported here GWAS results for the profiles of 65 FA traits and the fat percentage in Brown Swiss cows’ milk, including also lesser-studied FAs and/or those present in small concentrations. Genomic heritabilities exhibited a medium-high relationship with the genetic heritabilities estimated using a standard animal model (r = 0.70). Similarly, we found a moderate agreement between AWM column-wise correlations and the genetic correlations among FAs (r = 0.70)^9^. Several GWAS studies are available for bovine milk FA profiles in Holstein and Jersey populations^17–19,34,35^. However, only the main or most representative individual FAs or FA groups have generally been investigated. We found some correspondence with previous results, e.g., the presence of overlapping regions on BTA26 (~2.5–39.0 Mbp) for 14:1*c9* and 14:1 index^17,18,34^, on BTA13 (~41.40–68.40 Mbp) for 8:0 and SCFAs^17^, and on BTA5 for 18:1 index (~25.4–28.0 Mbp) and unsaturated FA (UFA) (84.1–104.70 Mbp)^35^. In particular, the region on BTA26 covers the known candidate gene *SCD1* (located at ~21.14 Mbp), confirming its notable influence on 14:1 index and consequently on 14:1*c9*. Indeed, although mammary SCD1 may act on several substrates (i.e. 14:0; 16:0, 17:0; 18:0; 18:1*t11*), 14:1 index has been considered the best proxy for SCD activity in the mammary gland, being 14:0 almost exclusively produced via *de novo* synthesis in the mammary gland^36^. Accordingly, the strongest association in the present study was found for 14:1 index (ARS-BFGL-NGS-32553, *P* = 1.36E-09) at 22.98 Mbp on BTA26, which is 1.84 Mb away from *SCD1*. Interestingly, we identified CNEs surrounding the marker Hapmap54104-rs29010930, which was located on BTA26 at 20.20 Mb, ~2 kb upstream of *CNNM1*, one of the top nodes in the reduced network. This marker was also detected by standard GWAS analysis and significantly associated to 14:0 (*P* = 2.16E-05; Supplementary Table S1). Because CNEs are now known to contribute 4.8% to 9.5% of the variability in the genome^37^, SNPs within this region may have important functional consequences on FA metabolism or synthesis in mammary gland. Indeed, recent findings suggest that CNEs are potentially involved in gene regulation, often encoding for enhancer elements, which may also be located far from their target gene^38,39^. Support for this hypothesis might be gathered by the results of GWAS analysis obtained after conditioning for Hapmap54104-rs29010930, which evidenced a large drop in h2 for 14:0 (from 0.075 to 0.017). The window of consecutive SNPs significantly associated with 14:1 index and 14:1*c9* in region 26b is very close to *SORBS1* (located at ~16.72–16.92 Mbp), which is involved in the regulation of insulin signalling in human adipose tissue^40^. Insulin acts as powerful regulator for the transcription of bovine *SCD* according to its pro-lipogenic role^41^. The ruminant mammary gland does not have a total requirement for insulin to activate lipogenesis but the lipogenic role of insulin signaling in the bovine mammary gland might acquire a biological meaning with the advancing of lactation and the increase in insulin sensitivity^42^. The *SORBS1* gene has also been previously reported as a potential candidate gene associated with bovine milk FA profile^35^. The same SNP at 104.71 Mbp on BTA5 (ARS-BFGL-NGS-99256) significantly affected PUFAs and n6 FAs (present work) and UFAs^35^. This SNP is located 4.46 Mbp from the *OLR1* gene, which can bind and degrade oxidized low-density lipoprotein and has already been identified as a candidate gene for milk fat composition^18,43^. Significant associations were detected on BTA2 (~129.05 Mbp), BTA25 (~32.65 Mbp) and BTA28 (~2.95 Mbp) for the CLA 18:2 *c9*,*t11*. A significant association for CLA on BTA2 was found also by^44^, although in a different position (98.2 Mbp). The SNP on BTA28 (ARS-BFGL-NGS-118662) was located 0.4 Mbp from *GNPAT*, which is involved in the lipid metabolic process and has previously been associated with Δ9-desaturase^44^. Other significant associations were discovered in close proximity of previously identified candidate genes^34,35^ such as *GH* (ARS-BFGL-NGS-102154), *LIPJ* and *LIPK* (ARS-BFGL-NGS-13746), *PRLR* (Hapmap26422-BTA-148751) and *NFKB2* (BTB-00933928) (Supplementary Table S1).

On the other hand, we also found some of our results to be inconsistent with previous studies. The most important differences were found on BTA14, where *DGAT1* (located at ~1,8Mbp) is recognised as influencing the profile of several FA traits, including RA, 14:1, 16:1, 16:0, 18:1*c9*, 14:1 index, 16:1 index, 18:1 index 18:2*c9*,*c12*, 18:3*c9*,*c12*,*c15* ^18,25^ and milk fat^19,45^. In the present study, we also found a significant association with the milk fat but at a different position (~26.00 Mbp). Other associations on BTA14 were with 18:1*t4* (~6.91 Mbp), 11:0 (~17.38 Mbp), 14:0 *iso* (~40.79 and 57.15 Mbp) and 15:0 *iso* (~40.79 and 58.85 Mbp). These differences can be attributed to the fact that *DGAT1* is nearly monomorphic in the Italian Brown Swiss^12,46^, therefore the observed associations might reflect the effect of other multiple genes. For instance, Hapmap25446-BTC-054694 was located ~0.3 Mbp from *CYP7A1* which catalyses a rate-limiting step in cholesterol catabolism^47^. Furthermore, a polymorphism on this gene has been shown to influence plasma lipids in humans^48^.

Our GWAS study did not detected SNPs associated to some of the well-known candidate genes for milk FA profile (e.g. *FASN*, *MGST1*). On the other hand, some other previously reported candidate genes were confirmed (e.g. *ORL1*, *GNPAT*, *GH*). GWAS power depends on sample size, effect size, causal allele frequency, and marker allele frequency and its correlation with the causal variant^49^. Some divergences with previous results might be attributed to several factors such as differences in the populations studied (breed, number of animals, environment and management conditions, physiological and metabolic factors, structure of the linkage disequilibrium among the genetic markers), and differences in the statistical model used for GWAS analysis and/or in the marker densities.

### Pathway and network analyses

Pathway and network analyses from GWAS data were performed to find both candidate regulatory sites and the most likely functional variants. We identified 12:0-related genes as uncovering the greatest number of pathways/GO terms, suggesting that 12:0 may play a key biological role in the control of milk fat composition. It is worth mentioning that the palm kernel oil, which is a rich source of 12:0, can be used as supplement in the feed industry. However, in Italy palm kernel oil is not regularly adopted as indirectly suggested by recent studies carried out in Northern Italy^50,51^. Concentrates might be integrated with lipids to increase the energy content of the diets but generally soybean, linseed and sunflower (as whole seeds, expellers or oil) are used.

**Figure 6:**
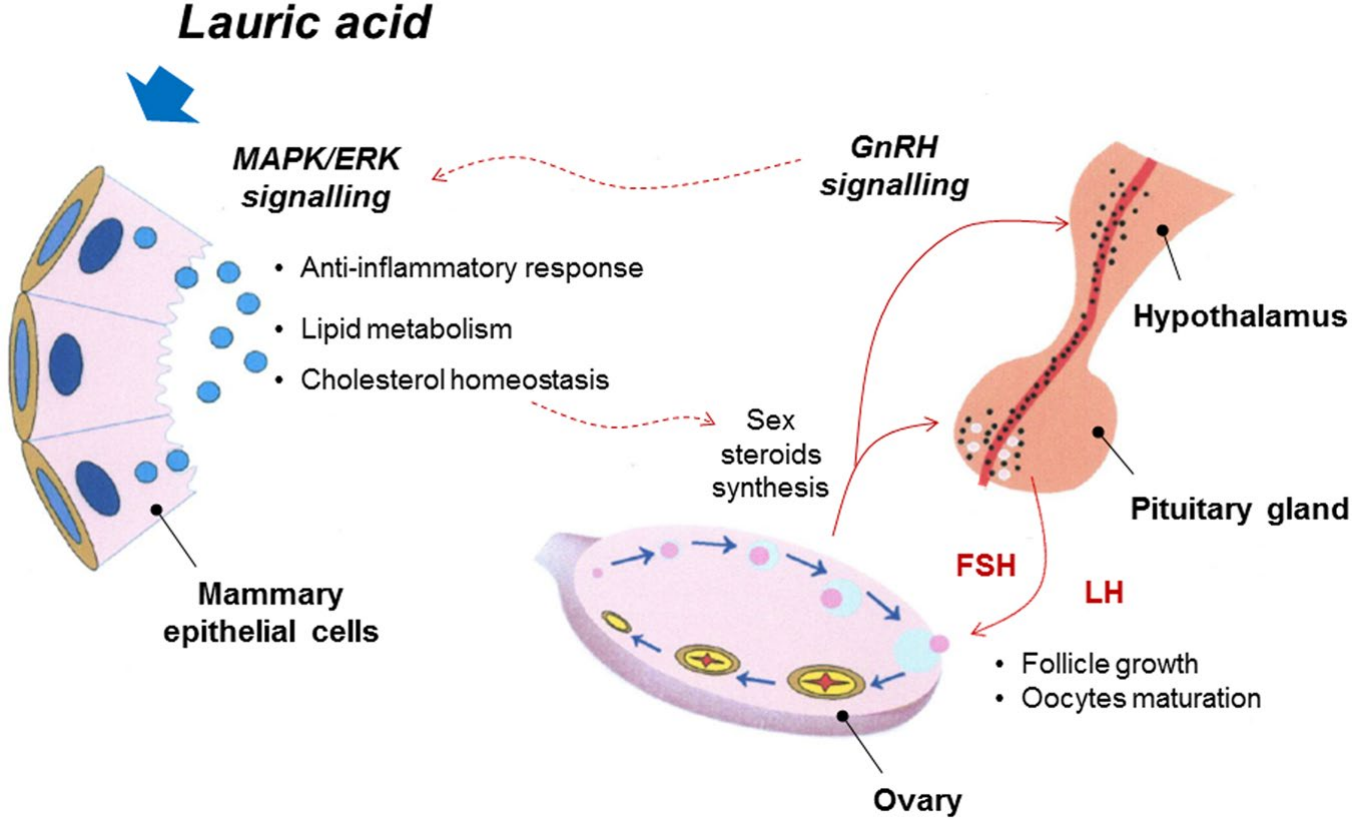
Potential roles of lauric acid in the bovine mammary gland. The Figure summarize the proposed roles for lauric acid based in the bovine mammary gland based on the results of functional and network analyses from the GWAS data. FSH: Follicle-stimulating hormone; LH: Luteinizing hormone.

The genes related to 12:0 were mostly involved in MAPK activity, ERK1 and ERK2 cascade and protein phosphorylation. The MAPK pathway has been found to be responsive to ApoA-1/ABCA1 activity in bovine mammary epithelial cells *in vitro* ^52^. Further, the presence of ABCA1 and ABCG1 has been detected in mammary epithelial cells and in the membrane of milk fat globules which suggest that these proteins might be involved in cholesterol exchange between mammary epithelial cells and milk^53^. In the ruminants, only a small part of cholesterol in milk seemed to be synthetised in the mammary gland while it is mainly derived from the blood uptake^54^. Genes related to the cholesterol synthesis are induced in the bovine liver after parturition^55^, suggesting that high levels of cholesterol are delivered by lipoproteins to the mammary gland^56^. Therefore, we might speculate that the significance of the association between 12:0 and alleles related to genes involved in MAPK pathway could be likely related to role of cholesterol in the fat globule membrane. Finally, capric and lauric acid have been also shown to have anti-bacterial and anti-inflammatory activities *in vitro* and in mouse model^57,58^. The anti-inflammatory effect seemed to be partially mediated by the inhibition of NF-κB activation and phosphorylation of MAPK^58^, suggesting a putative link between the immune signalling pathways and the mammary cells’ anti-inflammatory response. Activation of the GnRH pathways suggests a link between lauric acid, MAPK activity and cholesterol synthesis and release. The MAPK are also acknowledged as being involved in the transcriptional activation of a wide variety of genes regulating the biosynthesis and secretion of the gonadotropins, e.g., the luteinizing hormone (LH) and the follicle-stimulating hormone (FSH), both related to sex steroid hormone synthesis, follicle growth and oocyte maturation^59^. Cholesterol is also the precursor for the biosynthesis of steroid hormones, including sex steroids and corticosteroids^60^. The enrichment of pathways related to reproduction, e.g. oocyte meiosis, has been also previously associated to the milk profile of 12:0 in Danish Jersey^19^. All these novel putative biological roles for 12:0 in the bovine mammary gland are summarized in Fig. 6.

Pathway analyses of the set of genes included in the regulatory network further confirmed the relevance of alleles involved in MAPK signalling, cholesterol biosynthesis, hormonal signalling and reproduction. Overrepresentation of the “Oxytocin signalling pathway” may be explained by oxytocin (OXT) effects in the regulation of milk secretion from lactating mammary gland in mammals including ruminants^61^. Oxytocin was suggested to have direct effects on the mammary epithelium in mammals, in that it stimulates milk lipid secretion, although the mechanisms are still not clear^62,63^. Finally, OXT also plays an important role in many reproduction-related functions in female ruminant; accordingly, steroid hormones seemed to act as (indirect) regulators of the OXT-receptor signalling^64^.

### Identification of transcription factors within the regulatory network

Mining for transcription factors and their putative target genes is an important component of the system biology approach and allows transcriptional networks, which may have important regulatory functions for FA metabolism and synthesis, to be uncovered. The AWM approach allowed inferring gene interactions and computing networks based on the co-association patterns across phenotypes, using the PCIT algorithm which is based on partial correlations and information criteria. On the other hand, IPA provided relevant networks and up-stream regulators analysis based on the information deposited in the expert-annotated Ingenuity Knowledge Base. Integrating these approaches allowed to formulate molecular mechanistic hypotheses and identify upstream regulators likely responsible for phenotypic or functional outcomes.

The TF network analyses revealed that the best trio of TFs (*E2F3*, *KDM5A* and *BACH2*) presented co-association with a large number of genes involved in processes related to lipid and energy metabolism described in mammary tissue of ruminants, e.g., fatty acid β-oxidation^65^, PPAR/RXR activation^66^ and triacylglycerol biosynthesis^67^. This is of interest because manipulation of those TFs networks either directly (e.g., inhibiting or activating one or several TFs in the network) or indirectly (e.g., through selection) can lead to changes in milk FA synthesis. E2F3, one of the key regulators, is a cell cycle-related factor whose function in non-ruminants is associated with the regulation of adipocyte differentiation^68^ and lipid metabolism^69^. This TF exhibited co-association with 487 target genes playing a role in regulating lipid metabolism/transport and glucose metabolism (e.g., *DGKB*, *SCD5*, *ARNTL2*, *HMGCR*, *DAGLA*, *PLA2G4A*, *ACOXL* and *LPCAT2)*. For instance, the concentrations of *trans*-FA and CLA isomers in milk have been positively associated to *SCD5* expression in bovine mammary gland^70^; however, its substrate affinity and role in the mammary tissue are still unclear^71^. The second predicted key TF was *BACH2*, which was co-associated with 250 genes. The BACH2 functions as transcriptional repressor at the immune system level in human^72^; however, this gene has also been associated with intramuscular fat content in cattle^73^. Predicted target genes included lipid-related genes, such as *INSIG1*, *ABCG1*, *RORA*, *HMGA2*, *ESR1* and *ACSL3*. The insulin-induced gene 1 (INSIG1) regulates the responsiveness of SREBP1 and 2 processing via SCAP, thereby altering the rate of lipogenesis^22^. Indeed, changes in dietary lipid composition affect the expression of *INSIG1* in bovine mammary gland^74^. Furthermore, SNPs on *INSIG1* were significantly associated with differences in UFAs, SFAs and desaturation indices in bovine milk^11^. Acyl-CoA synthetase long-chain family member 3 (ACSL3) plays a role in the conversion of free LCFAs into fatty acyl-CoA esters controlling lipid biosynthesis and FA oxidation. Previous works have identified SNPs on *ACSL3*, that were associated with bovine milk FA composition^75,76^. The third predicted TF was *KDM5A*, which exhibited co-association with 269 target genes. The KDM5A functions as histone demethylases and contributes to co-repression of gene transcription. The KDM5A target genes include a large set of cell cycle regulators and genes involved in lipid metabolism, such as *LPIN1*, *ACACA*, *CHPT1*, *DGKG*, *GNPAT*, *PLD1* and *ADCY3*. The LPIN1 functions as a nuclear transcriptional co-activator to regulate the channeling of FAs toward milk triglyceride synthesis in the bovine mammary gland^77^, whereas ACACA catalyzes the carboxylation of acetyl-CoA to malonyl-CoA, which is the rate-limiting reaction in FA biosynthesis^78^. Polymorphisms on *LPIN*1 and *ACACA* have been recently associated to the bovine milk FA profile^14,16^.

These TF identified as being responsive to the milk FA profile might represent new targets for more detailed functional studies. However, it is worth mentioning that no direct evidence for *E2F3*, *KDM5A* and *BACH2* genes expression in bovine mammary gland is currently available^79^; therefore, investigation of their expression level in bovine mammary epithelial cells is needed to gain support for their potential role as regulators of milk FA profile at molecular level. On the other hand, in cow these TFs are expressed in tissues related to nervous system, i.e. hypothalamus (*E2F3*, *KDM5A* and *BACH3*) and brain (*E2F3*)^79^; it is well known that molecular pathways controlling FA metabolism are highly interconnected and therefore we might assume a putative relationship with FA even if these genes are not expressed in the mammary gland.

Current limitations for a more complete systems biology approach to milk FA regulation in dairy cows include a lack of additional “omics” data (e.g., metabolomics, proteomics, microRNAomics, epigenomics) and the incomplete genome annotation. Further, functional pathways available in livestock animals are still limited and most of the information is derived from human and animal models studies^28^. Consequently, the identification of competitive functional pathways is often challenging. Despite these limitations, our results show that genetic variants in the *E2F3*, *BACH2* and *KDM5A* genes probably play a key role in modulating bovine milk fat profile. Interestingly, these genes and many other lipid-related genes were not identified by standard GWAS approach, due to the stringent *P*-value threshold. The use of GWAS analysis coupled to network analysis allowed for a more holistic study of the multi-faceted control of fatty acid composition in milk. Even if some of our findings do not fully agree with previous GWAS studies in terms of (re)discovery of well-known candidate genes for bovine milk FA profile, the presented results are substantially coherent with biological processes and cellular functions implicated in the regulation of lipid metabolism in bovine mammary gland. Such information provides the basis for more detailed functional studies at the level of transcription factors and the subsequent effects on transcriptional networks. The new knowledge of the genetic control of milk FA composition could help in the development of selection strategies aimed at improving the quality of milk for human consumption. In particular, the information inferred from the present study might be incorporated as biological prior into prediction models such as BayesRC^80^ and GFBLUP^81^ to shed more light into the genetic basis of complex traits such as milk FA profile and to improve the accuracy of genomic prediction. On the other hand, the potential limitation of this approach relies on the need to underpin the actual role of the predicted gene regulators in the bovine mammary gland. Supporting evidences might be obtained by using co-expression analyses^82^, aiming to validate the predicted gene-gene interactions and to shed more light on the biological pathways driving variations in milk fat profile. It is likely that establishing a functional rationale underlying the importance of allelic variation and candidate genes for milk FA composition will become a major component of following up the genes emerging from GWAS.

## Methods

### Ethics statement

The cows included in this study belonged to commercial private herds and were not subjected to any invasive procedures. Milk and blood samples were previously collected during the routine milk recording coordinated by technicians working at the Breeder Association of Trento Province (Italy) and therefore authorized by a local authority.

### Animals, phenotypes and genotypes

Milk samples from 1,264 Italian Brown Swiss cows belonging to 85 herds located in Trento province in north-eastern Italy were collected once during the evening milking, as described in^83^. Herds were selected to represent different environments and dairy farming systems including feeding regimens (e.g. different amount and type of forage, amount of compound feed or type of ration). Details about dairy farming systems including animals feeding conditions are reported in^84^. Immediately after milking, the milk samples (without preservative) were refrigerated at 4 °C and transferred to the laboratory.

Analysis of the milk fat composition has been previously described^9^. In short, fat percentage was determined in individual milk samples using a MilkoScan FT6000 (Foss, Hillerød, Denmark) and analysis of the FA profile was performed using gas chromatography (GC) with 65 milk FA traits included (47 individual FAs, 13 FA groups and 5 desaturation indices).

The Illumina BovineSNP50 v.2 BeadChip (Illumina Inc., San Diego, CA) was used to genotype 1,152 cows (blood samples were not available for all phenotyped animals). Quality control excluded markers that did not satisfy the following criteria: (1) call rate > 95%, (2) minor allele frequency > 0.5%, and (3) no extreme deviation from Hardy-Weinberg proportions (*P* > 0.001, Bonferroni corrected). After this filtering step, 1,011 cows and 37,568 SNP were retained.

### Genome-wide association study

A single marker regression model was fitted for GWAS using the GenABEL package^85^ in the R environment and the GRAMMAR-GC (Genome wide Association using Mixed Model and Regression - Genomic Control) approach with the default function *gamma* ^86^. The GRAMMAR-GC procedure consists of 3 steps. Firstly, an additive polygenic model with a genomic relationship matrix is fitted. The polygenic model was:

1 $${\bf{y}}={\bf{X}}\beta +{\bf{a}}+{\bf{e}},$$

where **y** is a vector of milk FA traits; *β* is a vector with fixed effects of days in milk (classes of 30 days each), parity of the cow (classes 1, 2, 3, ≥4) and herd-date (n = 85); **X** is the incidence matrix that associates each observation to specific levels of the factors in *β*. The two random terms in the model were animal and the residuals, which were assumed to be normally distributed as ${\boldsymbol{a}}\, \sim N(0,{\bf{G}}{\sigma }_{g}^{2})$ and ${\boldsymbol{e}}\, \sim N(0,{\bf{I}}{\sigma }_{e}^{2})$, where **G** is the genomic relationship matrix, **I** is an identity matrix, and ${\sigma }_{g}^{2}$ and ${\sigma }_{e}^{2}$ are the additive genomic and residual variances, respectively. The **G** matrix was constructed in the GenABEL R package, where for a given pair of individuals i and j, the identical by state coefficients (${f}_{i,j}$) is calculated as:

2 $${f}_{i,j}=\frac{1}{N}{\sum }_{k}\frac{({x}_{i,k}-{p}_{k})\,\times \,({x}_{j,k}-{p}_{k})}{{p}_{k}\,\times (1-{p}_{k})}$$

where N is the number of markers used, ${x}_{i,k}$ is the genotype of the i^th^ individual at the k^th^ SNP (coded as 0, ½ and 1), ${p}_{k}$ is the frequency of the “+” allele and k = 1, …, N.

In the second step of GRAMMAR-GC, the residuals obtained in (1) are regressed on the SNP (single marker regression) to test for associations. In the last step, the Genomic Control (GC) approach corrects for conservativeness of the GRAMMAR procedure and estimates of the marker effects are obtained^87^. A *P*-value threshold of 5 × ${10}^{-5}$ was adopted to determine significant associations^88^. Manhattan plots were drawn using the R package qqman^89^. The variance explained by each SNP was calculated as 2pqa^2^, where p is the frequency of one allele, q = 1-p is the frequency of the second allele and a is the estimated additive genetic effect. A scan for genes around 1Mbp upstream-downstream from the significant SNP was performed using the Ensembl Bos taurus UMD3.1 database (http://www.ensembl.org/index.html).

Model (1) was also used to estimate variance components and the genomic heritability of the traits based on the genomic relationship matrix. Heritability was estimated as:

3 $${h}^{2}=\frac{{\sigma }_{g}^{2}}{{\sigma }_{g}^{2}+{\sigma }_{e}^{2}}$$

The proportion of the phenotypic variance explained by the SNPs included in the AWM was estimated using GenABEL and the previously described model. Firstly, a ***G*** matrix based only on the SNP s included in the AWM was constructed. Secondly, the same number of randomly selected SNPs was used to build 10,000 ***G*** matrices (10,000 replicates) and estimate the proportion of phenotypic variance explained by these randomly selected SNPs.

The r-squared statistic was chosen to predict the extent of LD using the R package LDheatmap^90^.

Conserved non-coding elements were explored using mVISTA, a web tool for comparative genomic analysis (http://genome.lbl.gov/vista/index.shtml), which allows sequences from multiple species to be compared and visualized with annotation information.

### Pathway analyses

Pathway analyses were performed to identify the biological mechanisms contributing to the milk fat profile, as previously detailed^29^. Briefly, the SNPs were divided into two categories “non-relevant” and “relevant” based on a nominal *P*-values < 0.05. By using a less stringent significance threshold (respect to the GWAS study), we aimed to capture the effect of less significant SNP which however can contribute to explain the variability for the investigated traits, possibly as part of organized pathways and/or biological processes. Then, the relevant SNPs were assigned to a gene if they were located within the gene or within a flanking region 15 kb up- and downstream it^91^, using the BiomaRt R package^92,93^ and the Ensembl *Bos taurus* UMD3.1 assembly as reference^94^. For functional annotation, the Kyoto Encyclopaedia of Genes and Genomes (KEGG)^95^ and the Gene Ontology (GO)^96^ databases were used to define pathways and functional categories associated to the gene sets. Only GO and KEGG terms with >10 and <1000 genes were included in the analyses to avoid testing broad or narrow functional categories. For each functional category, a Fisher’s exact test was applied to test for overrepresentation of significant gene sets. A false discovery rate (FDR) correction was used to control for false positives with the cut-off for significant enrichments set at FDR < 0.05. The gene-set enrichment analysis was performed using the Bioconductor package goseq in the R environment^31^.

### SNP co-association and network analyses

Along with the biological pathway analysis, SNP co-association and network analyses were carried out in order to detect key gene regulators, functional connections and networks of genes affecting the milk fat profile.

Given that many of the original annotations for the BovineSNP50 v.2 BeadChip (Illumina Inc., San Diego, CA) have been found to be incomplete, the Illumina BovineSNP50 v.3 Genotyping Beadchip annotation (available since June 2016 at http://www.illumina.com/products/by-type/microarray-kits/bovine-snp50.html) was used to re-assign the probes to new probe sets based on SNP position.

The AWM was built starting from the results of a GWAS analysis carried out without imposing a significance threshold. In particular, the AWM was constructed from two matrices that contained row-wise SNPs and column-wise phenotypes, as previously reported in detail^30,97^. Elements in the first matrix were equal to the *P* value of association for each SNP and phenotype, while in the second matrix corresponded to the SNP z-score standardised additive effect. Based on the results of the pathway analyses, which showed that the 12:0 FA was associated with the greatest number of overrepresented categories/pathways, the 12:0 FA was selected as the key phenotype and the associated SNPs (*P* ≤ 0.05) were included in the AWM. In the next step, dependency among phenotypes was explored by estimating the average number of other phenotypes (Ap) that were associated with these SNPs at the same *P-*value threshold (≤0.05) (Ap = 8). Subsequently, all SNPs that were associated with at least 8 phenotypes at *P* ≤ 0.05 were included in the AWM. In the next steps, the AWM was built following the procedure described by^30^, but only SNPs within genes or located close to intergenic SNPs (within 10 kb of the coding region) were selected. A distance of 10 kb was chosen because the probable size of the promoter region of a given gene is heterogeneous. In addition, to identify putative regulators, the TFs reported by^33^ and the microRNAs (miRNAs) that were mapped to the UMD3.1 bovine genome assembly (GenBank assembly accession: GCA_000003055.3) were also included in this analysis. The Pearson correlations obtained from pair-wise correlations of columns were computed and visualised as a clustering tree using the *hclust* R function^98^. The table-like structure of the AWM was used as input to the information theory (PCIT) algorithm, which uses a partial correlation in an information theory framework to ascertain significant gene–gene interactions (co-associations)^99^. To consider only the high-confidence gene co-associations determined by PCIT, those with correlation ≥|0.80| were retained, on the assumption that those genes have relevant biological significance for the key phenotype from which the AWM-PCIT was derived. The co-association network was automatically laid out using the organic layout algorithm in Cytoscape V2.7 (http://cytoscape.org). Network topological parameters and node centrality values were calculated using NetworkAnalyzer^100^ and CentiScape plugins^101^ to gain insights into the organisation and structure of the complex networks formed by the interacting molecules. In parallel, the list of co-associated genes was also fed into an Ingenuity Pathway Analysis (IPA, version 5.5; Ingenuity Systems, USA) to identify relevant categories of molecular functions, cellular components and biological processes. The IPA enabled us to identify (i) significantly overrepresented functional GO annotations, (ii) their over- or under-expression, and (iii) group-specific transcriptional networks. All listed or reconstructed cellular pathways were derived from the Ingenuity Knowledge Base which collects biological interactions and functional annotations derived from various experimental contexts and manually curated for accuracy from the literature and third-party databases. The IPA output a statistical assessment (based on a Fisher’s exact test) of the significance of representation for biological functions and signaling pathways (*P*-value < 0.05). The IPA computed networks and ranked them according to a statistical likelihood approach.

Once the TFs and their target genes to which they were potentially connected were identified in the AWM-derived network, an information lossless approach^102^ was used to identify the optimal subset of TFs spanning the majority of the network topology. Pathway and ontology analyses of the predicted target genes (co-associated with the best TF trio) were carried out using the Cytoscape plugin ClueGo^103^. The Benjamini & Hochberg correction for multiple testing was used with the cut-off for significant enrichment set at FDR < 0.05. The LASAGNA-Search 2.0 web tool^104^ was used to search for TFs binding site using matrices in the TRANSFAC public database and a *P*-value significance threshold of 0.001.

## Electronic supplementary material

Supplementary information

Supplementary Dataset 2

Supplementary Dataset 3

Supplementary Dataset 4

Supplementary Dataset 5

Supplementary Dataset 6
