# Supplementary material for: SNP co-association and network analyses identify E2F3, KDM5A and BACH2 as key regulators of the bovine milk fatty acid profile
Source: Sci Rep. 2017 Dec 11;7:17317. doi: 10.1038/s41598-017-17434-7 (PMC5725496; doi:10.1038/s41598-017-17434-7)
Supplement: Supplementary file 1 — Supplementary information [file 41598_2017_17434_MOESM1_ESM.docx]

**SNP co-association and network analyses identify E2F3, KDM5A and BACH2 as key regulators of the bovine milk fatty acid profile**

**Sara Pegolo**^1^***, Christos Dadousis**^1^**, Núria Mach**^2^**, Yuliaxis Ramayo-Caldas**^2,3^**, Marcello Mele**^4^**, Giuseppe Conte**^4^**, Giovanni Bittante**^1^, **Stefano Schiavon**^1^ **& Alessio Cecchinato**^1^

^1^Department of Agronomy, Food, Natural resources, Animals and Environment (DAFNAE), University of Padua, Viale dell’Università 16, 35020 Legnaro, Padua, Italy

^2^Animal Genetics and Integrative Biology unit (GABI), INRA, AgroParisTech, Université Paris-Saclay, 78350, Jouy-en-Josas, France

^3^Animal Breeding and Genetics Program, Institute for Research and Technology in Food and Agriculture (IRTA), Torre Marimon, Caldes de Montbui, 08140, Spain

^4^Department of Agriculture, Food and Environment, University of Pisa, Via del Borghetto 80, 56124 Pisa, Italy

*Corresponding author: [sara.pegolo@unipd.it](mailto:sara.pegolo@unipd.it)

**Supplementary Figure 1.** Hierarchical cluster analysis of the fatty acid traits analyzed in this study. SFA: saturated fatty acids; MUFA: mono-unsaturated fatty acids; PUFA: polyunsaturated fatty acids; SCFA: short-chain fatty acids; MCFA: medium-chain fatty acids; LCFA: long-chain fatty acids; BCFA: branched-chain fatty acids; OCFA: odd-chain fatty acids; RA: rumenic acid; VA: vaccenic acid.

**Supplementary Figure 2.** Comparison of genomic regions upstream CNNM1 across different species.

**Supplementary Table S1.** Complete list of the significant SNP obtained after GWAS analysis.

| **Trait** | **SNP** | **CHR** | **BP** | **P** | **LOG** | **effB** | **MAF** | **VSNP** | **Vp** | **Va** | **Vp%** | **Va%** |
| --- | --- | --- | --- | --- | --- | --- | --- | --- | --- | --- | --- | --- |
| 16:0iso | ARS-BFGL-NGS-102692 | 0 | 0 | 5.30E-06 | 5.275526 | 0.013312 | 0.455446 | 0.000088 | 0.00342 | 0.00051 | 2.57 | 17.24 |
| 18:2t11,c15 | BTB-00021257 | 0 | 0 | 3.22E-05 | 4.492724 | 0.005902 | 0.377756 | 0.000016 | 0.00086 | 0 | 1.91 | - |
| 16:1t9 | Hapmap43001-BTA-63377 | 0 | 0 | 4.90E-05 | 4.310127 | 0.005662 | 0.188614 | 0.000010 | 0.00055 | 0 | 1.78 | - |
| 13:0 | DPI-28 | 1 | 37046480 | 3.75E-05 | 4.426319 | 0.007596 | 0.209693 | 0.000019 | 0.00101 | 4.00E-05 | 1.89 | 47.75 |
| 18:1c12 | Hapmap49149-BTA-39529 | 1 | 81271586 | 2.83E-05 | 4.548249 | -0.01477 | 0.206726 | 0.000072 | 0.00341 | 0.00057 | 2.10 | 12.56 |
| ID14:1 | ARS-BFGL-NGS-23253 | 1 | 89900842 | 4.00E-05 | 4.397672 | 0.006866 | 0.060831 | 0.000005 | 0.00025 | 8.00E-05 | 2.16 | 6.74 |
| 17:1c9 | BTB-01748272 | 1 | 92171349 | 1.37E-05 | 4.862614 | 0.063679 | 0.008433 | 0.000068 | 0.00349 | 0 | 1.94 | - |
| n6/n3 | BTA-49368-no-rs | 1 | 121093829 | 4.04E-05 | 4.394136 | 0.110425 | 0.4825 | 0.006089 | 0.3148 | 0.03414 | 1.93 | 17.84 |
| BCFA | ARS-BFGL-NGS-42512 | 1 | 138452988 | 4.42E-05 | 4.354401 | 0.068171 | 0.133037 | 0.001072 | 0.05135 | 0.01345 | 2.09 | 7.97 |
| 14:0iso | ARS-BFGL-NGS-42512 | 1 | 138452988 | 1.55E-05 | 4.808843 | 0.011155 | 0.133037 | 0.000029 | 0.00129 | 0.00017 | 2.22 | 16.88 |
| 16:0iso | ARS-BFGL-NGS-42512 | 1 | 138452988 | 1.20E-07 | 6.919414 | 0.022537 | 0.133037 | 0.000117 | 0.00342 | 0.00051 | 3.43 | 22.97 |
| n6/n3 | BTB-01839901 | 1 | 140996730 | 1.34E-05 | 4.871695 | 0.211339 | 0.088702 | 0.007221 | 0.3148 | 0.03414 | 2.29 | 21.15 |
| 18:1t11 | ARS-BFGL-NGS-97531 | 1 | 148969868 | 4.23E-06 | 5.373514 | 0.091776 | 0.068744 | 0.001078 | 0.04323 | 0.00637 | 2.49 | 16.93 |
| TFA | ARS-BFGL-NGS-97531 | 1 | 148969868 | 7.05E-06 | 5.151902 | 0.137984 | 0.068744 | 0.002438 | 0.09901 | 0.01928 | 2.46 | 12.64 |
| TFA18:1 | ARS-BFGL-NGS-97531 | 1 | 148969868 | 8.44E-06 | 5.07375 | 0.134115 | 0.068744 | 0.002303 | 0.09535 | 0.01844 | 2.42 | 12.49 |
| 18:2c9,t11 | ARS-BFGL-NGS-77983 | 2 | 129053865 | 6.94E-06 | 5.158868 | 0.030231 | 0.274529 | 0.000364 | 0.01494 | 0.00276 | 2.44 | 13.19 |
| OCFA | ARS-BFGL-NGS-56131 | 2 | 130511453 | 3.00E-06 | 5.522963 | 0.064751 | 0.2 | 0.001342 | 0.05093 | 0.00512 | 2.63 | 26.20 |
| n6/n3 | ARS-BFGL-NGS-100336 | 3 | 13148505 | 3.06E-05 | 4.514524 | 0.646241 | 0.007929 | 0.006570 | 0.3148 | 0.03414 | 2.09 | 19.24 |
| 17:1c9 | ARS-BFGL-NGS-108225 | 3 | 45572779 | 1.09E-05 | 4.961877 | 0.079794 | 0.005446 | 0.000069 | 0.00349 | 0 | 1.98 | - |
| 17:1c9 | Hapmap59096-rs29024776 | 3 | 49335664 | 5.73E-06 | 5.241674 | 0.059848 | 0.010386 | 0.000074 | 0.00349 | 0 | 2.11 | - |
| 17:1c9 | ARS-BFGL-NGS-119366 | 3 | 56959267 | 8.50E-06 | 5.070782 | 0.07734 | 0.005935 | 0.000071 | 0.00349 | 0 | 2.02 | - |
| 17:1c9 | ARS-BFGL-NGS-69251 | 3 | 57515766 | 3.49E-06 | 5.457403 | 0.072202 | 0.007418 | 0.000077 | 0.00349 | 0 | 2.20 | - |
| 11:0 | BTB-00135284 | 3 | 72743814 | 1.13E-07 | 6.946868 | 0.037214 | 0.008408 | 0.000023 | 7.00E-04 | 4.00E-05 | 3.30 | 57.75 |
| 15:0 | BTB-00135284 | 3 | 72743814 | 1.06E-05 | 4.972798 | 0.202424 | 0.008408 | 0.000683 | 0.02875 | 0.00271 | 2.38 | 25.21 |
| 24:0 | BTB-00135284 | 3 | 72743814 | 2.95E-06 | 5.530448 | 0.018515 | 0.008408 | 0.000006 | 0.00023 | 1.00E-05 | 2.49 | 57.20 |
| OCFA | BTB-00135284 | 3 | 72743814 | 1.56E-05 | 4.807245 | 0.265653 | 0.008408 | 0.001177 | 0.05093 | 0.00512 | 2.31 | 22.98 |
| 10:1c9 | ARS-BFGL-NGS-34260 | 3 | 78170557 | 1.73E-05 | 4.763032 | -0.02825 | 0.058358 | 0.000088 | 0.00388 | 0.00101 | 2.26 | 8.68 |
| 17:0iso | ARS-BFGL-NGS-98739 | 3 | 118567045 | 3.09E-06 | 5.509625 | -0.01298 | 0.344156 | 0.000076 | 0.00315 | 0.00031 | 2.42 | 24.55 |
| 17:0ante | BTA-69827-no-rs | 3 | 118592298 | 1.73E-05 | 4.762304 | -0.01193 | 0.378833 | 0.000067 | 0.00315 | 0.00031 | 2.13 | 21.61 |
| 17:0iso | ARS-BFGL-NGS-10050 | 3 | 119686073 | 8.99E-06 | 5.046119 | -0.01271 | 0.330693 | 0.000072 | 0.00315 | 0.00031 | 2.27 | 23.06 |
| 17:0iso | BTB-01730472 | 3 | 120283544 | 1.28E-06 | 5.891152 | 0.014125 | 0.336806 | 0.000089 | 0.00315 | 0.00031 | 2.83 | 28.74 |
| 17:0iso | ARS-BFGL-NGS-76298 | 3 | 120446774 | 4.78E-05 | 4.320575 | 0.011187 | 0.413519 | 0.000061 | 0.00315 | 0.00031 | 1.93 | 19.58 |
| 17:0iso | ARS-BFGL-NGS-68307 | 3 | 121203750 | 1.46E-05 | 4.834631 | -0.01259 | 0.306886 | 0.000068 | 0.00315 | 0.00031 | 2.14 | 21.77 |
| 24:0 | Hapmap49725-BTA-72716 | 4 | 25155275 | 4.90E-06 | 5.309459 | 0.004958 | 0.128091 | 0.000005 | 0.00023 | 1.00E-05 | 2.39 | 54.90 |
| n3 | ARS-BFGL-NGS-43812 | 4 | 84080999 | 1.49E-05 | 4.827127 | 0.07158 | 0.028884 | 0.000287 | 0.01292 | 0.00257 | 2.22 | 11.18 |
| 11:0 | Hapmap49738-BTA-75486 | 5 | 10335741 | 1.85E-05 | 4.733164 | -0.00591 | 0.315529 | 0.000015 | 7.00E-04 | 4.00E-05 | 2.16 | 37.75 |
| OCFA | UA-IFASA-4222 | 5 | 10511028 | 1.04E-05 | 4.982574 | -0.05844 | 0.227723 | 0.001201 | 0.05093 | 0.00512 | 2.36 | 23.46 |
| OCFA | BTA-119598-no-rs | 5 | 10626452 | 2.85E-05 | 4.544829 | -0.05977 | 0.190406 | 0.001101 | 0.05093 | 0.00512 | 2.16 | 21.51 |
| 15:0 | BTA-23621-no-rs | 5 | 10735432 | 3.01E-05 | 4.521431 | -0.06135 | 0.091494 | 0.000626 | 0.02875 | 0.00271 | 2.18 | 23.09 |
| OCFA | BTA-23621-no-rs | 5 | 10735432 | 1.02E-05 | 4.990493 | -0.08678 | 0.091494 | 0.001252 | 0.05093 | 0.00512 | 2.46 | 24.45 |
| 15:0 | ARS-BFGL-NGS-117548 | 5 | 10788597 | 3.82E-05 | 4.418481 | -0.06159 | 0.087043 | 0.000603 | 0.02875 | 0.00271 | 2.10 | 22.25 |
| OCFA | ARS-BFGL-NGS-117548 | 5 | 10788597 | 1.10E-05 | 4.958951 | -0.08795 | 0.087043 | 0.001229 | 0.05093 | 0.00512 | 2.41 | 24.01 |
| ID18:1 | Hapmap30002-BTA-142983 | 5 | 26876852 | 7.18E-06 | 5.143868 | -0.05245 | 0.008911 | 0.000049 | 0.00147 | 0.00038 | 3.31 | 12.79 |
| 18:0 | ARS-BFGL-NGS-22065 | 5 | 41695035 | 3.66E-05 | 4.437072 | -0.37928 | 0.187004 | 0.043741 | 2.0853 | 0.47143 | 2.10 | 9.28 |
| 18:0 | BTA-73516-no-rs | 5 | 48752237 | 4.24E-05 | 4.372835 | 0.306841 | 0.334817 | 0.041938 | 2.0853 | 0.47143 | 2.01 | 8.90 |
| 10:0 | Hapmap49290-BTA-74411 | 5 | 84086946 | 1.01E-05 | 4.995992 | -0.11794 | 0.266073 | 0.005433 | 0.22442 | 0.04423 | 2.42 | 12.28 |
| 12:0 | Hapmap49290-BTA-74411 | 5 | 84086946 | 8.80E-06 | 5.055305 | -0.14164 | 0.266073 | 0.007836 | 0.3123 | 0.07335 | 2.51 | 10.68 |
| LCFA | Hapmap49290-BTA-74411 | 5 | 84086946 | 2.93E-05 | 4.533614 | 0.813592 | 0.266073 | 0.258522 | 12.2796 | 1.49812 | 2.11 | 17.26 |
| MCFA | Hapmap49290-BTA-74411 | 5 | 84086946 | 4.91E-05 | 4.308847 | -0.75336 | 0.266073 | 0.221660 | 10.8027 | 2.09261 | 2.05 | 10.59 |
| MUFA | Hapmap49290-BTA-74411 | 5 | 84086946 | 4.11E-05 | 4.385981 | 0.599395 | 0.266073 | 0.140317 | 7.02701 | 0.73651 | 2.00 | 19.05 |
| SFA | Hapmap49290-BTA-74411 | 5 | 84086946 | 1.13E-05 | 4.947475 | -0.72607 | 0.266073 | 0.205895 | 8.76784 | 1.36579 | 2.35 | 15.08 |
| 10:0 | Hapmap39359-BTA-89420 | 5 | 84374008 | 4.71E-05 | 4.326775 | -0.10668 | 0.278437 | 0.004573 | 0.22442 | 0.04423 | 2.04 | 10.34 |
| 12:0 | Hapmap39359-BTA-89420 | 5 | 84374008 | 4.58E-06 | 5.339298 | -0.14334 | 0.278437 | 0.008255 | 0.3123 | 0.07335 | 2.64 | 11.25 |
| 10:0 | ARS-BFGL-NGS-103280 | 5 | 84523411 | 1.56E-05 | 4.808218 | -0.10915 | 0.306627 | 0.005066 | 0.22442 | 0.04423 | 2.26 | 11.45 |
| 12:0 | ARS-BFGL-NGS-103280 | 5 | 84523411 | 2.00E-05 | 4.699917 | -0.12855 | 0.306627 | 0.007027 | 0.3123 | 0.07335 | 2.25 | 9.58 |
| 10:0 | ARS-BFGL-NGS-72008 | 5 | 85160180 | 2.37E-05 | 4.625948 | -0.1027 | 0.351137 | 0.004806 | 0.22442 | 0.04423 | 2.14 | 10.87 |
| 12:0 | ARS-BFGL-NGS-72008 | 5 | 85160180 | 2.66E-06 | 5.575334 | -0.1361 | 0.351137 | 0.008440 | 0.3123 | 0.07335 | 2.70 | 11.51 |
| 10:0 | ARS-BFGL-NGS-39308 | 5 | 85288739 | 3.58E-05 | 4.446439 | -0.11183 | 0.240356 | 0.004566 | 0.22442 | 0.04423 | 2.03 | 10.32 |
| PUFA | ARS-BFGL-NGS-99256 | 5 | 104714350 | 1.64E-05 | 4.786332 | 0.105195 | 0.372383 | 0.005173 | 0.21604 | 0.05462 | 2.39 | 9.47 |
| n6 | ARS-BFGL-NGS-99256 | 5 | 104714350 | 3.92E-05 | 4.406564 | 0.074579 | 0.372383 | 0.002600 | 0.12285 | 0.02048 | 2.12 | 12.69 |
| 20:1c9 | ARS-BFGL-NGS-91167 | 5 | 108721301 | 3.15E-05 | 4.501245 | 0.009671 | 0.105159 | 0.000018 | 0.00086 | 5.00E-05 | 2.05 | 35.20 |
| 20:4c5,c8,c11,c14 | Hapmap46514-BTA-122322 | 6 | 1091047 | 5.72E-06 | 5.242419 | 0.053908 | 0.006036 | 0.000035 | 0.00131 | 0.00029 | 2.66 | 12.03 |
| ID_CLA | Hapmap46514-BTA-122322 | 6 | 1091047 | 1.11E-07 | 6.954276 | 0.073912 | 0.006036 | 0.000066 | 0.00186 | 2.00E-04 | 3.53 | 32.80 |
| 18:1t16 | ARS-BFGL-NGS-118959 | 6 | 21829670 | 6.17E-06 | 5.209456 | 0.016163 | 0.176063 | 0.000076 | 0.00311 | 0.00028 | 2.44 | 27.07 |
| 18:1t16 | ARS-BFGL-NGS-100050 | 6 | 23148412 | 1.28E-05 | 4.893502 | 0.015908 | 0.153314 | 0.000066 | 0.00311 | 0.00028 | 2.11 | 23.46 |
| 17:0ante | Hapmap23862-BTC-069949 | 6 | 40530400 | 1.06E-05 | 4.972773 | 0.090786 | 0.00544 | 0.000089 | 0.00361 | 0.00052 | 2.47 | 17.15 |
| 18:2t11,c15 | Hapmap38352-BTA-76628 | 6 | 70865694 | 2.80E-05 | 4.552934 | 0.015233 | 0.037624 | 0.000017 | 0.00086 | 0 | 1.95 | - |
| 18:1t6-t8 | BTA-76070-no-rs | 6 | 78598487 | 2.31E-05 | 4.635911 | 0.066704 | 0.005952 | 0.000053 | 0.00223 | 0.00018 | 2.36 | 29.28 |
| BCFA | ARS-BFGL-NGS-106506 | 7 | 8491850 | 2.34E-05 | 4.630851 | -0.06553 | 0.168327 | 0.001202 | 0.05135 | 0.01345 | 2.34 | 8.94 |
| 15:0ante | ARS-BFGL-NGS-106506 | 7 | 8491850 | 3.51E-07 | 6.454979 | -0.02502 | 0.168327 | 0.000175 | 0.00493 | 0.0011 | 3.55 | 15.93 |
| 18:1c12 | ARS-BFGL-NGS-27096 | 7 | 34158112 | 6.38E-06 | 5.194877 | 0.040587 | 0.027201 | 0.000087 | 0.00341 | 0.00057 | 2.56 | 15.30 |
| fat | BTB-01848865 | 7 | 87008149 | 7.80E-06 | 5.107844 | 0.229897 | 0.134558 | 0.014644 | 0.551145 | 0.056091 | 2.66 | 26.11 |
| 16:0 | BTB-01862398 | 7 | 89421646 | 3.05E-05 | 4.515956 | 2.766707 | 0.009425 | 0.142925 | 6.65641 | 2.29735 | 2.15 | 6.22 |
| 18:1t16 | Hapmap40047-BTA-119117 | 8 | 3663959 | 2.53E-09 | 8.59629 | 0.094884 | 0.006924 | 0.000124 | 0.00311 | 0.00028 | 3.98 | 44.22 |
| 15:0ante | ARS-BFGL-NGS-103495 | 8 | 9393962 | 4.83E-05 | 4.316243 | -0.01611 | 0.278437 | 0.000104 | 0.00493 | 0.0011 | 2.12 | 9.49 |
| 16:0 | BTA-109900-no-rs | 8 | 22479821 | 3.51E-05 | 4.454806 | 0.624601 | 0.238614 | 0.141754 | 6.65641 | 2.29735 | 2.13 | 6.17 |
| SFA | Hapmap31882-BTA-80969 | 8 | 36781224 | 3.24E-05 | 4.489897 | -0.90844 | 0.133168 | 0.190528 | 8.76784 | 1.36579 | 2.17 | 13.95 |
| 24:0 | ARS-BFGL-NGS-66921 | 8 | 63901386 | 2.82E-05 | 4.549044 | -0.00523 | 0.08457 | 0.000004 | 0.00023 | 1.00E-05 | 1.84 | 42.40 |
| 20:3c8,c11,c14 | ARS-BFGL-NGS-79292 | 8 | 76353681 | 2.39E-05 | 4.62124 | 0.020877 | 0.034653 | 0.000029 | 0.00141 | 7.00E-05 | 2.07 | 41.71 |
| 13:0 | ARS-BFGL-NGS-25285 | 8 | 86501009 | 1.58E-05 | 4.801563 | 0.044869 | 0.005935 | 0.000024 | 0.00101 | 4.00E-05 | 2.36 | 59.50 |
| 18:1c12 | BTB-00372235 | 8 | 102361784 | 2.43E-05 | 4.614033 | 0.019216 | 0.116222 | 0.000076 | 0.00341 | 0.00057 | 2.23 | 13.32 |
| LCFA | Hapmap57174-rs29021038 | 8 | 109112032 | 1.59E-05 | 4.799286 | 2.436084 | 0.025248 | 0.292097 | 12.2796 | 1.49812 | 2.38 | 19.50 |
| MCFA | Hapmap57174-rs29021038 | 8 | 109112032 | 2.60E-05 | 4.584294 | -2.26303 | 0.025248 | 0.252072 | 10.8027 | 2.09261 | 2.33 | 12.05 |
| MUFA | Hapmap57174-rs29021038 | 8 | 109112032 | 3.87E-05 | 4.412259 | 1.737501 | 0.025248 | 0.148591 | 7.02701 | 0.73651 | 2.11 | 20.18 |
| SFA | Hapmap57174-rs29021038 | 8 | 109112032 | 2.11E-05 | 4.675594 | -2.03466 | 0.025248 | 0.203764 | 8.76784 | 1.36579 | 2.32 | 14.92 |
| 17:1c9 | ARS-BFGL-NGS-17233 | 8 | 110602353 | 2.18E-05 | 4.662418 | 0.052509 | 0.012376 | 0.000067 | 0.00349 | 0 | 1.93 | - |
| 14:0iso | ARS-BFGL-NGS-93995 | 9 | 10453697 | 8.76E-06 | 5.057732 | 0.008229 | 0.361881 | 0.000031 | 0.00129 | 0.00017 | 2.43 | 18.41 |
| 14:0iso | ARS-BFGL-NGS-15511 | 9 | 10492302 | 8.27E-06 | 5.082557 | 0.008247 | 0.361249 | 0.000031 | 0.00129 | 0.00017 | 2.43 | 18.47 |
| 24:0 | Hapmap38633-BTA-83140 | 9 | 10858927 | 4.69E-06 | 5.328743 | -0.00333 | 0.422046 | 0.000005 | 0.00023 | 1.00E-05 | 2.36 | 54.20 |
| 20:1c9 | ARS-BFGL-NGS-82987 | 9 | 13141454 | 3.67E-05 | 4.435107 | -0.02683 | 0.011397 | 0.000016 | 0.00086 | 5.00E-05 | 1.88 | 32.40 |
| ID18:1 | BTB-00389124 | 9 | 35036949 | 3.71E-06 | 5.431161 | -0.04882 | 0.008911 | 0.000042 | 0.00147 | 0.00038 | 2.86 | 11.08 |
| 22:4c7,c10,c13,c16 | BTB-00396747 | 9 | 61292190 | 4.02E-05 | 4.396071 | 0.004289 | 0.109344 | 0.000004 | 0.00018 | 1.00E-05 | 1.99 | 35.80 |
| fat | BTB-00403297 | 9 | 91346194 | 1.23E-06 | 5.909911 | 0.17342 | 0.419095 | 0.011828 | 0.551145 | 0.056091 | 2.15 | 21.09 |
| 17:0 | ARS-BFGL-NGS-25581 | 9 | 97050334 | 3.00E-05 | 4.523259 | -0.01546 | 0.342235 | 0.000108 | 0.00502 | 0.00045 | 2.14 | 23.93 |
| 16:0 | ARS-BFGL-NGS-72947 | 9 | 102301291 | 9.79E-06 | 5.0092 | 0.568773 | 0.472664 | 0.161268 | 6.65641 | 2.29735 | 2.42 | 7.02 |
| 20:0 | ARS-BFGL-NGS-34445 | 9 | 104529625 | 2.98E-05 | 4.526114 | 0.006874 | 0.379482 | 0.000022 | 0.0011 | 0.00012 | 2.03 | 18.58 |
| TFA | ARS-BFGL-NGS-34445 | 9 | 104529625 | 4.43E-05 | 4.353658 | 0.06562 | 0.379482 | 0.002028 | 0.09901 | 0.01928 | 2.05 | 10.52 |
| TFA18:1 | ARS-BFGL-NGS-34445 | 9 | 104529625 | 2.99E-05 | 4.523836 | 0.065751 | 0.379482 | 0.002036 | 0.09535 | 0.01844 | 2.14 | 11.04 |
| SCFA | BTB-00415258 | 10 | 28680745 | 3.29E-05 | 4.483279 | 0.26112 | 0.249258 | 0.025518 | 1.29287 | 0.1562 | 1.97 | 16.34 |
| ID10:1 | BTB-00424023 | 10 | 51775341 | 3.79E-05 | 4.421262 | -0.00346 | 0.308605 | 0.000005 | 0.00024 | 5.00E-05 | 2.13 | 10.22 |
| 15:0 | BTA-111053-no-rs | 10 | 98541920 | 5.78E-06 | 5.238287 | -0.03865 | 0.40099 | 0.000718 | 0.02875 | 0.00271 | 2.50 | 26.48 |
| 11:0 | Hapmap36552-SCAFFOLD185127_16827 | 11 | 2767247 | 1.99E-05 | 4.702057 | 0.030019 | 0.008964 | 0.000016 | 7.00E-04 | 4.00E-05 | 2.29 | 40.00 |
| SCFA | ARS-BFGL-NGS-37630 | 11 | 46590323 | 4.74E-05 | 4.32441 | -0.28171 | 0.19802 | 0.025207 | 1.29287 | 0.1562 | 1.95 | 16.14 |
| 20:3c8,c11,c14 | ARS-BFGL-NGS-22364 | 11 | 72900738 | 1.57E-05 | 4.804167 | 0.018845 | 0.043521 | 0.000030 | 0.00141 | 7.00E-05 | 2.10 | 42.29 |
| 20:1c9 | ARS-BFGL-NGS-13252 | 12 | 81184160 | 2.29E-05 | 4.640194 | 0.007061 | 0.2364 | 0.000018 | 0.00086 | 5.00E-05 | 2.09 | 36.00 |
| 18:1t6-t8 | ARS-BFGL-NGS-113366 | 12 | 82564341 | 4.09E-06 | 5.388526 | 0.029616 | 0.030198 | 0.000051 | 0.00223 | 0.00018 | 2.30 | 28.56 |
| 18:1c12 | ARS-BFGL-NGS-93056 | 13 | 8880814 | 4.36E-05 | 4.36053 | -0.01339 | 0.273492 | 0.000071 | 0.00341 | 0.00057 | 2.09 | 12.51 |
| 15:0 | Hapmap51705-BTA-24478 | 13 | 21822462 | 1.58E-05 | 4.801526 | 0.099589 | 0.034192 | 0.000655 | 0.02875 | 0.00271 | 2.28 | 24.17 |
| OCFA | Hapmap51705-BTA-24478 | 13 | 21822462 | 1.71E-05 | 4.766674 | 0.13268 | 0.034192 | 0.001163 | 0.05093 | 0.00512 | 2.28 | 22.71 |
| SCFA | Hapmap61089-rs29014590 | 13 | 42070556 | 3.21E-05 | 4.493486 | -1.45732 | 0.006487 | 0.027375 | 1.29287 | 0.1562 | 2.12 | 17.53 |
| 8:0 | ARS-BFGL-NGS-74106 | 13 | 42148059 | 2.71E-05 | 4.567642 | -0.2113 | 0.007426 | 0.000658 | 0.03069 | 0.00467 | 2.14 | 14.09 |
| SCFA | ARS-BFGL-NGS-74106 | 13 | 42148059 | 4.71E-06 | 5.327294 | -1.4843 | 0.007426 | 0.032477 | 1.29287 | 0.1562 | 2.51 | 20.79 |
| 10:1c9 | Hapmap47251-BTA-32677 | 13 | 42230721 | 8.29E-06 | 5.081547 | -0.05936 | 0.014342 | 0.000100 | 0.00388 | 0.00101 | 2.57 | 9.86 |
| SCFA | Hapmap47251-BTA-32677 | 13 | 42230721 | 9.13E-06 | 5.039324 | -1.04259 | 0.014342 | 0.030732 | 1.29287 | 0.1562 | 2.38 | 19.68 |
| SCFA | ARS-BFGL-NGS-31452 | 13 | 42257575 | 7.98E-06 | 5.098004 | -1.27931 | 0.009901 | 0.032088 | 1.29287 | 0.1562 | 2.48 | 20.54 |
| 15:0ante | ARS-BFGL-NGS-57335 | 13 | 42927726 | 1.76E-05 | 4.755555 | 0.082838 | 0.009397 | 0.000128 | 0.00493 | 0.0011 | 2.59 | 11.61 |
| 22:5c7,c10,c13,c16,c19 | ARS-BFGL-NGS-26401 | 13 | 56128139 | 1.43E-05 | 4.845209 | 0.016169 | 0.022066 | 0.000011 | 0.00053 | 3.00E-05 | 2.13 | 37.67 |
| 22:5c7,c10,c13,c16,c19 | BTB-00534445 | 13 | 60198826 | 1.72E-05 | 4.764401 | 0.027636 | 0.007426 | 0.000011 | 0.00053 | 3.00E-05 | 2.13 | 37.67 |
| 17:0 | ARS-BFGL-NGS-36046 | 13 | 79523868 | 6.76E-06 | 5.170176 | -0.01561 | 0.432903 | 0.000120 | 0.00502 | 0.00045 | 2.38 | 26.58 |
| 17:0ante | ARS-BFGL-NGS-19988 | 13 | 83018263 | 2.82E-05 | 4.550074 | 0.026492 | 0.055391 | 0.000073 | 0.00361 | 0.00052 | 2.03 | 14.12 |
| 18:1t4 | Hapmap25183-BTC-049425 | 14 | 6910008 | 3.60E-05 | 4.44358 | -0.00312 | 0.338645 | 0.000004 | 0.00023 | 0 | 1.90 | - |
| 11:0 | ARS-BFGL-NGS-114730 | 14 | 17378950 | 2.82E-05 | 4.549636 | 0.01843 | 0.016484 | 0.000011 | 7.00E-04 | 4.00E-05 | 1.57 | 27.50 |
| fat | Hapmap25446-BTC-054694 | 14 | 26003598 | 8.25E-06 | 5.083499 | 0.159304 | 0.457962 | 0.012310 | 0.551145 | 0.056091 | 2.23 | 21.95 |
| 14:0iso | Hapmap50929-BTA-28833 | 14 | 40785938 | 6.74E-06 | 5.171122 | 0.032114 | 0.015347 | 0.000031 | 0.00129 | 0.00017 | 2.42 | 18.35 |
| 15:0iso | Hapmap50929-BTA-28833 | 14 | 40785938 | 2.38E-06 | 5.622622 | 0.039957 | 0.015347 | 0.000048 | 0.00185 | 0.00018 | 2.61 | 26.83 |
| 14:0iso | BTA-35074-no-rs | 14 | 57145781 | 3.82E-05 | 4.418124 | 0.025219 | 0.020317 | 0.000025 | 0.00129 | 0.00017 | 1.96 | 14.88 |
| 15:0iso | ARS-BFGL-NGS-18262 | 14 | 58848872 | 3.71E-05 | 4.431099 | 0.019113 | 0.052032 | 0.000036 | 0.00185 | 0.00018 | 1.95 | 20.00 |
| 22:4c7,c10,c13,c16 | ARS-BFGL-NGS-89820 | 15 | 9041018 | 4.94E-05 | 4.306503 | -0.00272 | 0.376371 | 0.000003 | 0.00018 | 1.00E-05 | 1.92 | 34.60 |
| 18:1c9 | Hapmap49882-BTA-121007 | 15 | 22960231 | 4.63E-05 | 4.334548 | -0.49487 | 0.481701 | 0.122286 | 6.16806 | 0.43828 | 1.98 | 27.90 |
| LCFA | Hapmap49882-BTA-121007 | 15 | 22960231 | 4.02E-05 | 4.396257 | -0.71727 | 0.481701 | 0.256893 | 12.2796 | 1.49812 | 2.09 | 17.15 |
| 17:0 | ARS-BFGL-NGS-100131 | 15 | 23154872 | 4.93E-05 | 4.307544 | -0.01477 | 0.44604 | 0.000108 | 0.00502 | 0.00045 | 2.15 | 23.97 |
| 10:1c9 | ARS-BFGL-BAC-27778 | 15 | 41043816 | 1.11E-05 | 4.954637 | -0.09562 | 0.006048 | 0.000110 | 0.00388 | 0.00101 | 2.83 | 10.88 |
| 14:1c9 | ARS-BFGL-BAC-27778 | 15 | 41043816 | 3.38E-05 | 4.471624 | -0.3407 | 0.006048 | 0.001396 | 0.0529 | 0.01511 | 2.64 | 9.24 |
| ID10:1 | ARS-BFGL-BAC-27778 | 15 | 41043816 | 1.38E-06 | 5.859087 | -0.02589 | 0.006048 | 0.000008 | 0.00024 | 5.00E-05 | 3.36 | 16.12 |
| 18:1t10 | Hapmap43354-BTA-77081 | 15 | 70284345 | 1.33E-05 | 4.875298 | -0.0135 | 0.409001 | 0.000088 | 0.00395 | 0.00029 | 2.23 | 30.41 |
| 15:0 | BTB-01487766 | 15 | 70793253 | 2.42E-05 | 4.615684 | -0.048 | 0.166172 | 0.000638 | 0.02875 | 0.00271 | 2.22 | 23.56 |
| fat | ARS-BFGL-NGS-87853 | 16 | 30262349 | 1.74E-05 | 4.759812 | 0.195292 | 0.191889 | 0.012599 | 0.551145 | 0.056091 | 2.29 | 22.46 |
| n3 | Hapmap41467-BTA-18750 | 16 | 34021608 | 2.74E-05 | 4.561554 | 0.027248 | 0.231913 | 0.000265 | 0.01292 | 0.00257 | 2.05 | 10.29 |
| 11:0 | BTA-38719-no-rs | 16 | 37229378 | 1.20E-05 | 4.920601 | 0.008917 | 0.11194 | 0.000016 | 7.00E-04 | 4.00E-05 | 2.26 | 39.50 |
| n3 | ARS-BFGL-NGS-77903 | 16 | 48694547 | 3.13E-05 | 4.504659 | 0.03431 | 0.133168 | 0.000272 | 0.01292 | 0.00257 | 2.10 | 10.58 |
| 14:0iso | BTA-105815-no-rs | 16 | 79192647 | 2.38E-05 | 4.623992 | 0.0079 | 0.324083 | 0.000027 | 0.00129 | 0.00017 | 2.12 | 16.06 |
| 18:3c9,c12,c15 | Hapmap47504-BTA-111690 | 17 | 11231535 | 8.10E-06 | 5.091314 | 0.088733 | 0.01731 | 0.000268 | 0.0104 | 0.00227 | 2.58 | 11.80 |
| n3 | Hapmap47504-BTA-111690 | 17 | 11231535 | 8.47E-06 | 5.071864 | 0.098785 | 0.01731 | 0.000332 | 0.01292 | 0.00257 | 2.57 | 12.92 |
| 18:1t11 | Hapmap42781-BTA-105847 | 17 | 20593969 | 2.79E-05 | 4.554451 | -0.04555 | 0.324324 | 0.000909 | 0.04323 | 0.00637 | 2.10 | 14.28 |
| 18:1t4 | Hapmap41708-BTA-99722 | 17 | 61624831 | 2.60E-05 | 4.585328 | 0.013512 | 0.011869 | 0.000004 | 0.00023 | 0 | 1.86 | - |
| 24:0 | ARS-BFGL-NGS-13495 | 17 | 69267462 | 3.78E-05 | 4.422926 | 0.005891 | 0.070722 | 0.000005 | 0.00023 | 1.00E-05 | 1.98 | 45.60 |
| 20:1c9 | ARS-BFGL-NGS-116944 | 18 | 53071113 | 4.84E-05 | 4.314977 | 0.00878 | 0.126858 | 0.000017 | 0.00086 | 5.00E-05 | 1.99 | 34.20 |
| TFA | ARS-BFGL-NGS-53296 | 19 | 7875822 | 4.32E-05 | 4.364568 | 0.065743 | 0.448071 | 0.002138 | 0.09901 | 0.01928 | 2.16 | 11.09 |
| 18:1t11 | ARS-BFGL-NGS-107289 | 19 | 10305065 | 4.67E-05 | 4.330329 | 0.060747 | 0.126117 | 0.000813 | 0.04323 | 0.00637 | 1.88 | 12.77 |
| TFA | ARS-BFGL-NGS-107289 | 19 | 10305065 | 5.83E-06 | 5.234108 | 0.10402 | 0.126117 | 0.002385 | 0.09901 | 0.01928 | 2.41 | 12.37 |
| TFA18:1 | ARS-BFGL-NGS-107289 | 19 | 10305065 | 6.34E-06 | 5.198101 | 0.101592 | 0.126117 | 0.002275 | 0.09535 | 0.01844 | 2.39 | 12.34 |
| 22:4c7,c10,c13,c16 | ARS-BFGL-NGS-20183 | 19 | 38365974 | 2.08E-05 | 4.682361 | 0.002872 | 0.390208 | 0.000004 | 0.00018 | 1.00E-05 | 2.18 | 39.30 |
| 22:4c7,c10,c13,c16 | UA-IFASA-8986 | 19 | 39201774 | 3.25E-05 | 4.488474 | -0.00271 | 0.426311 | 0.000004 | 0.00018 | 1.00E-05 | 2.00 | 36.00 |
| 14:0 | ARS-BFGL-NGS-102154 | 19 | 49329111 | 4.58E-05 | 4.339503 | -0.54983 | 0.039565 | 0.022975 | 1.18345 | 0.08885 | 1.94 | 25.86 |
| OCFA | UA-IFASA-6210 | 19 | 59715027 | 1.36E-05 | 4.866219 | 0.075509 | 0.106541 | 0.001085 | 0.05093 | 0.00512 | 2.13 | 21.20 |
| 18:1c9 | BTB-00765879 | 19 | 61585054 | 4.03E-05 | 4.395055 | 1.452085 | 0.029208 | 0.119575 | 6.16806 | 0.43828 | 1.94 | 27.28 |
| 10:1c9 | ARS-BFGL-NGS-31267 | 19 | 62113537 | 3.78E-05 | 4.422268 | -0.01367 | 0.354599 | 0.000086 | 0.00388 | 0.00101 | 2.20 | 8.47 |
| 20:1c9 | ARS-BFGL-NGS-31729 | 19 | 62829939 | 8.28E-06 | 5.082008 | 0.006425 | 0.416584 | 0.000020 | 0.00086 | 5.00E-05 | 2.34 | 40.20 |
| ID18:1 | BTB-00780480 | 20 | 36757600 | 7.31E-06 | 5.135919 | -0.01488 | 0.09545 | 0.000038 | 0.00147 | 0.00038 | 2.61 | 10.08 |
| ID18:1 | ARS-BFGL-NGS-5430 | 20 | 37838938 | 1.13E-06 | 5.94502 | -0.0176 | 0.075668 | 0.000043 | 0.00147 | 0.00038 | 2.95 | 11.39 |
| 17:0 | Hapmap26422-BTA-148751 | 20 | 40754324 | 4.66E-05 | 4.331408 | 0.072295 | 0.009891 | 0.000102 | 0.00502 | 0.00045 | 2.04 | 22.75 |
| 18:2c9,c12 | BTB-00793802 | 20 | 61402228 | 4.44E-05 | 4.352615 | -0.06927 | 0.335149 | 0.002138 | 0.10285 | 0.01681 | 2.08 | 12.72 |
| 24:0 | ARS-BFGL-NGS-99194 | 20 | 61764939 | 2.80E-05 | 4.553497 | 0.005599 | 0.076347 | 0.000004 | 0.00023 | 1.00E-05 | 1.92 | 44.20 |
| 10:1c9 | ARS-BFGL-NGS-103163 | 20 | 68933034 | 9.33E-06 | 5.030069 | -0.01349 | 0.380693 | 0.000086 | 0.00388 | 0.00101 | 2.21 | 8.50 |
| 14:1c9 | ARS-BFGL-NGS-60835 | 20 | 71271028 | 2.56E-05 | 4.591808 | -0.0497 | 0.4199 | 0.001203 | 0.0529 | 0.01511 | 2.27 | 7.96 |
| 22:5c7,c10,c13,c16,c19 | Hapmap39215-BTA-105710 | 21 | 4706119 | 4.06E-05 | 4.391463 | 0.005034 | 0.268051 | 0.000010 | 0.00053 | 3.00E-05 | 1.88 | 33.13 |
| n6 | ARS-BFGL-NGS-119424 | 21 | 12684633 | 2.76E-05 | 4.558773 | 0.232814 | 0.024233 | 0.002563 | 0.12285 | 0.02048 | 2.09 | 12.52 |
| 6:0 | Hapmap59970-rs29026939 | 21 | 42413671 | 1.75E-05 | 4.757734 | 0.058098 | 0.278437 | 0.001356 | 0.06327 | 0.00549 | 2.14 | 24.70 |
| SCFA | Hapmap59970-rs29026939 | 21 | 42413671 | 3.48E-05 | 4.458637 | 0.254632 | 0.278437 | 0.026053 | 1.29287 | 0.1562 | 2.02 | 16.68 |
| BCFA | ARS-BFGL-NGS-37805 | 21 | 63985298 | 4.12E-05 | 4.385512 | -0.06505 | 0.147525 | 0.001064 | 0.05135 | 0.01345 | 2.07 | 7.91 |
| BCFA | ARS-BFGL-NGS-43652 | 21 | 64085350 | 1.67E-06 | 5.776408 | -0.07496 | 0.154419 | 0.001468 | 0.05135 | 0.01345 | 2.86 | 10.91 |
| 16:0iso | ARS-BFGL-NGS-43652 | 21 | 64085350 | 2.30E-05 | 4.6383 | -0.01691 | 0.154419 | 0.000075 | 0.00342 | 0.00051 | 2.18 | 14.65 |
| 18:3c9,t11,c15 | ARS-BFGL-NGS-19546 | 22 | 21547900 | 3.13E-05 | 4.504135 | 0.017946 | 0.009397 | 0.000006 | 0.00033 | 0 | 1.82 | - |
| 17:1c9 | ARS-BFGL-NGS-20317 | 22 | 29854903 | 3.10E-06 | 5.50895 | 0.042586 | 0.024233 | 0.000086 | 0.00349 | 0 | 2.46 | - |
| 6:0 | ARS-BFGL-NGS-8294 | 22 | 46781645 | 6.60E-06 | 5.180549 | 0.091044 | 0.100396 | 0.001497 | 0.06327 | 0.00549 | 2.37 | 27.27 |
| 6:0 | ARS-BFGL-NGS-2876 | 22 | 46810135 | 2.42E-05 | 4.615368 | 0.085763 | 0.09801 | 0.001300 | 0.06327 | 0.00549 | 2.06 | 23.69 |
| 6:0 | ARS-BFGL-NGS-100411 | 22 | 47365483 | 9.68E-06 | 5.014162 | 0.091335 | 0.092977 | 0.001407 | 0.06327 | 0.00549 | 2.22 | 25.63 |
| n6/n3 | ARS-BFGL-NGS-82789 | 22 | 58458470 | 2.00E-06 | 5.697917 | 0.216208 | 0.104352 | 0.008738 | 0.3148 | 0.03414 | 2.78 | 25.59 |
| 18:1t16 | Hapmap40178-BTA-55802 | 23 | 21748514 | 2.00E-05 | 4.69828 | 0.012306 | 0.361083 | 0.000070 | 0.00311 | 0.00028 | 2.25 | 24.96 |
| 15:0 | ARS-BFGL-NGS-109297 | 23 | 36139914 | 8.29E-06 | 5.081571 | 0.070333 | 0.074 | 0.000678 | 0.02875 | 0.00271 | 2.36 | 25.02 |
| OCFA | ARS-BFGL-NGS-109297 | 23 | 36139914 | 4.74E-05 | 4.324446 | 0.085864 | 0.074 | 0.001010 | 0.05093 | 0.00512 | 1.98 | 19.73 |
| 14:0iso | ARS-BFGL-NGS-19695 | 24 | 14841074 | 4.20E-05 | 4.376326 | -0.02456 | 0.021782 | 0.000026 | 0.00129 | 0.00017 | 1.99 | 15.12 |
| 20:1c9 | ARS-BFGL-NGS-104621 | 24 | 48334377 | 2.11E-05 | 4.676521 | 0.008913 | 0.126113 | 0.000018 | 0.00086 | 5.00E-05 | 2.03 | 35.00 |
| 16:0iso | Hapmap29767-BTC-015734 | 25 | 2084697 | 1.68E-05 | 4.775088 | -0.02026 | 0.097718 | 0.000072 | 0.00342 | 0.00051 | 2.12 | 14.20 |
| ID18:1 | ARS-BFGL-NGS-108964 | 25 | 4566007 | 4.53E-06 | 5.343528 | -0.03216 | 0.018793 | 0.000038 | 0.00147 | 0.00038 | 2.59 | 10.03 |
| 17:0iso | Hapmap43893-BTA-60736 | 25 | 9862811 | 2.47E-05 | 4.607255 | -0.01491 | 0.18719 | 0.000068 | 0.00315 | 0.00031 | 2.15 | 21.84 |
| 18:2c9,t11 | BTA-118965-no-rs | 25 | 32648759 | 3.14E-07 | 6.503588 | 0.045806 | 0.142347 | 0.000512 | 0.01494 | 0.00276 | 3.43 | 18.56 |
| PUFA | BTA-118965-no-rs | 25 | 32648759 | 2.02E-05 | 4.694805 | 0.146205 | 0.142347 | 0.005219 | 0.21604 | 0.05462 | 2.42 | 9.56 |
| ID10:1 | ARS-BFGL-NGS-13746 | 26 | 9866940 | 9.98E-06 | 5.000776 | -0.00528 | 0.125618 | 0.000006 | 0.00024 | 5.00E-05 | 2.55 | 12.26 |
| ID14:1 | ARS-BFGL-NGS-13746 | 26 | 9866940 | 3.05E-06 | 5.515731 | -0.00592 | 0.125618 | 0.000008 | 0.00025 | 8.00E-05 | 3.08 | 9.61 |
| ID14:1 | Hapmap26713-BTA-150121 | 26 | 14654938 | 2.71E-06 | 5.567741 | -0.00669 | 0.091494 | 0.000007 | 0.00025 | 8.00E-05 | 2.98 | 9.30 |
| 14:1c9 | ARS-BFGL-NGS-21545 | 26 | 14975265 | 1.24E-06 | 5.906552 | -0.08888 | 0.116683 | 0.001628 | 0.0529 | 0.01511 | 3.08 | 10.78 |
| ID14:1 | ARS-BFGL-NGS-21545 | 26 | 14975265 | 2.35E-07 | 6.629244 | -0.00657 | 0.116683 | 0.000009 | 0.00025 | 8.00E-05 | 3.56 | 11.13 |
| 14:1c9 | ARS-BFGL-NGS-43432 | 26 | 15336560 | 2.99E-06 | 5.524334 | -0.06255 | 0.284158 | 0.001592 | 0.0529 | 0.01511 | 3.01 | 10.53 |
| ID10:1 | ARS-BFGL-NGS-43432 | 26 | 15336560 | 1.07E-05 | 4.970655 | -0.00386 | 0.284158 | 0.000006 | 0.00024 | 5.00E-05 | 2.52 | 12.10 |
| ID14:1 | ARS-BFGL-NGS-43432 | 26 | 15336560 | 1.67E-08 | 7.778419 | -0.00524 | 0.284158 | 0.000011 | 0.00025 | 8.00E-05 | 4.48 | 14.00 |
| 14:1c9 | ARS-BFGL-NGS-104463 | 26 | 16708402 | 3.91E-05 | 4.408333 | -0.05072 | 0.347525 | 0.001167 | 0.0529 | 0.01511 | 2.21 | 7.72 |
| ID14:1 | ARS-BFGL-NGS-104463 | 26 | 16708402 | 2.31E-07 | 6.635484 | -0.00442 | 0.347525 | 0.000009 | 0.00025 | 8.00E-05 | 3.55 | 11.10 |
| 14:1c9 | Hapmap44394-BTA-62134 | 26 | 18169859 | 6.47E-06 | 5.189341 | 0.059465 | 0.301193 | 0.001489 | 0.0529 | 0.01511 | 2.81 | 9.85 |
| ID14:1 | Hapmap44394-BTA-62134 | 26 | 18169859 | 2.33E-06 | 5.632791 | 0.004321 | 0.301193 | 0.000008 | 0.00025 | 8.00E-05 | 3.14 | 9.83 |
| ID14:1 | Hapmap46848-BTA-60788 | 26 | 18848144 | 1.09E-05 | 4.963353 | -0.00522 | 0.152324 | 0.000007 | 0.00025 | 8.00E-05 | 2.81 | 8.79 |
| 14:1c9 | Hapmap40565-BTA-62157 | 26 | 19263805 | 1.48E-05 | 4.829345 | 0.055609 | 0.316832 | 0.001339 | 0.0529 | 0.01511 | 2.53 | 8.86 |
| ID14:1 | Hapmap40565-BTA-62157 | 26 | 19263805 | 2.52E-05 | 4.598075 | 0.003752 | 0.316832 | 0.000006 | 0.00025 | 8.00E-05 | 2.44 | 7.61 |
| 10:0 | BTA-62171-no-rs | 26 | 19507012 | 1.13E-05 | 4.948073 | -0.11727 | 0.267096 | 0.005384 | 0.22442 | 0.04423 | 2.40 | 12.17 |
| 12:0 | BTA-62171-no-rs | 26 | 19507012 | 2.29E-06 | 5.640197 | -0.15061 | 0.267096 | 0.008880 | 0.3123 | 0.07335 | 2.84 | 12.11 |
| 14:0 | ARS-BFGL-NGS-115980 | 26 | 20065602 | 3.41E-06 | 5.466671 | 0.25335 | 0.341476 | 0.028867 | 1.18345 | 0.08885 | 2.44 | 32.49 |
| 14:0 | Hapmap54104-rs29010930 | 26 | 20203731 | 2.16E-05 | 4.666345 | 0.228334 | 0.484174 | 0.026042 | 1.18345 | 0.08885 | 2.20 | 29.31 |
| 14:0 | BTB-00931586 | 26 | 21409429 | 1.01E-05 | 4.997155 | 0.314428 | 0.166172 | 0.027397 | 1.18345 | 0.08885 | 2.32 | 30.84 |
| 18:1c9 | BTB-00931586 | 26 | 21409429 | 3.23E-05 | 4.490912 | -0.66701 | 0.166172 | 0.123291 | 6.16806 | 0.43828 | 2.00 | 28.13 |
| ID10:1 | BTB-00931586 | 26 | 21409429 | 2.33E-05 | 4.633168 | -0.00442 | 0.166172 | 0.000005 | 0.00024 | 5.00E-05 | 2.25 | 10.82 |
| ID14:1 | BTB-00931586 | 26 | 21409429 | 8.07E-08 | 7.092896 | -0.00594 | 0.166172 | 0.000010 | 0.00025 | 8.00E-05 | 3.92 | 12.24 |
| MUFA | BTB-00931586 | 26 | 21409429 | 4.22E-06 | 5.374664 | -0.79679 | 0.166172 | 0.175935 | 7.02701 | 0.73651 | 2.50 | 23.89 |
| 14:1c9 | BTB-00933928 | 26 | 22446047 | 3.83E-05 | 4.416714 | -0.08098 | 0.098286 | 0.001162 | 0.0529 | 0.01511 | 2.20 | 7.69 |
| ID10:1 | BTB-00933928 | 26 | 22446047 | 3.20E-06 | 5.494943 | -0.00598 | 0.098286 | 0.000006 | 0.00024 | 5.00E-05 | 2.64 | 12.66 |
| ID14:1 | BTB-00933928 | 26 | 22446047 | 1.95E-08 | 7.709669 | -0.00767 | 0.098286 | 0.000010 | 0.00025 | 8.00E-05 | 4.16 | 13.00 |
| 14:1c9 | ARS-BFGL-NGS-39823 | 26 | 22951431 | 7.52E-06 | 5.12369 | -0.09378 | 0.086548 | 0.001391 | 0.0529 | 0.01511 | 2.63 | 9.20 |
| ID10:1 | ARS-BFGL-NGS-39823 | 26 | 22951431 | 7.41E-06 | 5.130103 | -0.00614 | 0.086548 | 0.000006 | 0.00024 | 5.00E-05 | 2.48 | 11.92 |
| 16:0iso | ARS-BFGL-NGS-39823 | 26 | 22951431 | 3.46E-09 | 8.460597 | -0.00858 | 0.086548 | 0.000012 | 0.00025 | 8.00E-05 | 4.64 | 14.50 |
| 14:1c9 | ARS-BFGL-NGS-32553 | 26 | 22977848 | 6.69E-06 | 5.174846 | -0.0875 | 0.103465 | 0.001420 | 0.0529 | 0.01511 | 2.68 | 9.40 |
| ID10:1 | ARS-BFGL-NGS-32553 | 26 | 22977848 | 8.27E-06 | 5.082486 | -0.00567 | 0.103465 | 0.000006 | 0.00024 | 5.00E-05 | 2.48 | 11.90 |
| ID14:1 | ARS-BFGL-NGS-32553 | 26 | 22977848 | 1.36E-09 | 8.865574 | -0.00817 | 0.103465 | 0.000012 | 0.00025 | 8.00E-05 | 4.96 | 15.50 |
| 14:1c9 | ARS-BFGL-NGS-118712 | 26 | 25088146 | 3.36E-06 | 5.473618 | -0.06955 | 0.194554 | 0.001516 | 0.0529 | 0.01511 | 2.87 | 10.03 |
| ID14:1 | ARS-BFGL-NGS-118712 | 26 | 25088146 | 9.05E-09 | 8.043126 | -0.00597 | 0.194554 | 0.000011 | 0.00025 | 8.00E-05 | 4.48 | 14.00 |
| 14:1c9 | ARS-BFGL-NGS-58864 | 26 | 25144793 | 1.48E-05 | 4.830206 | 0.053508 | 0.356786 | 0.001314 | 0.0529 | 0.01511 | 2.48 | 8.70 |
| ID14:1 | ARS-BFGL-NGS-58864 | 26 | 25144793 | 2.47E-05 | 4.607041 | 0.003614 | 0.356786 | 0.000006 | 0.00025 | 8.00E-05 | 2.40 | 7.49 |
| 14:1c9 | UA-IFASA-4715 | 26 | 25314352 | 1.37E-05 | 4.861994 | -0.05646 | 0.298298 | 0.001334 | 0.0529 | 0.01511 | 2.52 | 8.83 |
| ID14:1 | UA-IFASA-4715 | 26 | 25314352 | 1.44E-06 | 5.840932 | -0.00435 | 0.298298 | 0.000008 | 0.00025 | 8.00E-05 | 3.18 | 9.93 |
| 14:1c9 | ARS-BFGL-NGS-65546 | 26 | 26296160 | 1.74E-05 | 4.760142 | -0.05538 | 0.290307 | 0.001264 | 0.0529 | 0.01511 | 2.39 | 8.36 |
| ID14:1 | ARS-BFGL-NGS-65546 | 26 | 26296160 | 8.74E-06 | 5.05841 | -0.00398 | 0.290307 | 0.000007 | 0.00025 | 8.00E-05 | 2.60 | 8.14 |
| ID14:1 | ARS-BFGL-NGS-117380 | 26 | 29261121 | 2.98E-05 | 4.52645 | -0.00434 | 0.190927 | 0.000006 | 0.00025 | 8.00E-05 | 2.32 | 7.26 |
| ID14:1 | ARS-BFGL-NGS-114723 | 26 | 31577458 | 1.61E-05 | 4.793601 | -0.00365 | 0.347181 | 0.000006 | 0.00025 | 8.00E-05 | 2.42 | 7.55 |
| fat | ARS-BFGL-NGS-87845 | 27 | 42118037 | 6.56E-07 | 6.183345 | 0.558217 | 0.02666 | 0.016172 | 0.551145 | 0.056091 | 2.93 | 28.83 |
| 18:2c9,t11 | ARS-BFGL-NGS-118662 | 28 | 2947166 | 2.52E-05 | 4.597754 | -0.10669 | 0.013875 | 0.000312 | 0.01494 | 0.00276 | 2.09 | 11.29 |
| 20:4c5,c8,c11,c14 | ARS-BFGL-NGS-67720 | 29 | 17876279 | 4.39E-05 | 4.357572 | 0.015118 | 0.057978 | 0.000025 | 0.00131 | 0.00029 | 1.91 | 8.62 |
| 14:0iso | BTA-65012-no-rs | 29 | 19966479 | 4.60E-06 | 5.337014 | 0.013478 | 0.096439 | 0.000032 | 0.00129 | 0.00017 | 2.46 | 18.65 |
| n6/n3 | ARS-BFGL-NGS-114570 | 29 | 25759091 | 2.79E-05 | 4.554455 | 0.142044 | 0.207221 | 0.006629 | 0.3148 | 0.03414 | 2.11 | 19.42 |

effB: SNP effect; LOG: log(base10) P; MAF: minor allele frequency; V_SNP_: SNP variance calculated as
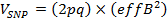


Vp: phenotypic variance; Va: additive genetic variance; Vp%= percentage of phenotypic variance explained by SNP; Va%= percentage of additive genetic variance explained by SNP
